# From Sensorimotor Inhibition to Freudian Repression: Insights from Psychosis Applied to Neurosis

**DOI:** 10.3389/fpsyg.2012.00452

**Published:** 2012-11-05

**Authors:** Ariane Bazan

**Affiliations:** ^1^Centre de Recherche en Psychologie Clinique, Psychopathologie et Psychosomatique, Faculté des Sciences Psychologiques et de l’Education, Université Libre de Bruxelles (ULB)Brussels, Belgium

**Keywords:** inhibition, repression, sensorimotor, Freud, efference copy, psychosis, unconscious, Lacan

## Abstract

First, three case studies are presented of psychotic patients having in common an inability to hold something down or out. In line with other theories on psychosis, we propose that a key change is at the efference copy system. Going back to Freud’s mental apparatus, we propose that the messages of discharge of the motor neurons, mobilized to direct perception, also called “indications of reality,” are equivalent to the modern efference copies. With this key, the reading of the cases is coherent with the psychodynamic understanding of psychosis, being a downplay of secondary processes, and consequently, a dominance of primary processes. Moreover, putting together the sensorimotor idea of a failure of efference copy-mediated inhibition with the psychoanalytic idea of a failing repression in psychosis, the hypothesis emerges that the attenuation enabled by the efference copy dynamics is, in some instances, the physiological instantiation of repression. Second, we applied this idea to the mental organization in neurosis. Indeed, the efference copy-mediated attenuation is thought to be the mechanism through which sustained activation of an intention, without reaching it – i.e., inhibition of an action – gives rise to mental imagery. Therefore, as inhibition is needed for any targeted action or for normal language understanding, acting in the world, or processing language, structurally induces mental imagery, constituting a subjective unconscious mental reality. Repression is a special instance of inhibition for emotionally threatening stimuli. These stimuli require stronger inhibition, leaving (the attenuation of) the motor intentions totally unanswered, in order to radically prevent execution which would lead to development of excess affect. This inhibition, then, yields a specific type of motor imagery, called “phantoms,” which induce mental preoccupation, as well as symptoms which, especially through their form, refer to the repressed motor fragments.

« Enlightenment rarely given to mortals has been given to me», says

Schreber (1903, p. 167)^1^.

## Faltering Inhibition in Psychosis

### Hervé and the constitution of an exterior perception space

#### Hervé

When I meet Hervé, he is 45 and he is at the psychiatric center of Beernem in Belgium^2^. Hervé is the only son of his parents who are respectively 57 (father) and 33 (mother) at his birth. His mother’s father had returned gravely traumatized from World War I. At the time of Hervé’s birth, he had already hanged himself after a history of violence against his wife and daughters. Hervé: “He beat his wife and was imprisoned. He was aggressive. He had six daughters and one son. My mother was beaten. He had sex with his daughters. He was mad, he was in the war of 14–18.” Hervé’s mother, the eldest, had two children from incestuous abuse by her father: a son who died age 5 from a kidney disease and a daughter, who died at 45 from cerebral hemorrhage. Hervé has not known the son but lived together with his half-sister, 15 years his elder and mentally handicapped^3^. Hervé’s problems start around the age of 13, he starts counting back- and forward. When somewhat older, seeing his father beat his mother, Hervé himself beats his father but “I beat mother most.” Hervé has frequent aggressive outbursts and at 18 is admitted at the psychiatric hospital. At 22, his father dies. At 29 and 30 he does several suicide attempts.

One of Hervé’s major difficulties is that, often, when he directs a glance to the world, the perception of this world invades him. He complains of penetrating sensations, people and things sticking onto his body, onto his skin, of people walking through him, of things penetrating him. In other words, he has great difficulty to create an experience of distance between him and his percepts. To restore a bearable relation to the world, he has to physically move back and forth around his visual targets: he frequently backs up on his steps, to move forward again; he opens doors by opening them partially, then closing them again partially, then reopening them, etc.; he does and undoes repeatedly, either completely or partially, some of his gestures, both in the forward and in the backward direction.

Specifically, one chain of events is painful to him: suddenly moving people or things in the world cause a “fizz” or a “pinching feeling” on his retina, which then constitutes the unique and direct cause of an “undesirable image.” These undesirable images are often of an incisive and penetrating content, such as described on the following note which he keeps in his coat^4^:

“11-09-1995. In Dymfna. That dirty coat of fat, basement slices. A needle in the eye. The disintegration of my photo-apparatus when G. C. went by the closet in the first living. Sucking out my eye. 28-11-‘95 A penis through my knife 28-11-1995. My pectoral muscle torn when P. L went through the corridor. Around that time transferred to St. Cornelius. My viscera extirpated by C. C. at the old laundry house on 21-12-’97. 21-11-98. When the occupational therapist went to the bookbinding place, Lieve C. instead of Koen C. When J. D. went from the place where the opener hangs to his chair. My balls unhooked or melted. When the fire chief went to the farm. My colon ruptured a little. When M. D. went from the toilet to the outside. My balls extirpated.^5^”

To undo the undesirable image, the moving target is asked to undo the movement: he asks people around him to back up, to undo in backward direction what they did in the first place. When they agree to do so, he watches the scene fixedly, firmly closes his eye, and holds this pose for a second, before looking up again. In case they do not submit to his request, he remains pursued by the painful image. Places where he has been, are thus occupied with an accumulation of as-yet-undone undesirable images, which continue to haunt him.

#### The constitution of an exterior perception space

We were struck by the similarities between Hervé’s experience and the way the constitution of perception has been described in sensorimotor terminology. In a sensorimotor framework, perception is not a passive event but a summary of succession of actions and sensations: it is the access to a law connecting the performed actions to their sensory consequences (the “law of sensorimotor contingency,” O’Regan and Noe, 2001). For example, the philosophy and cognitive science professor Lenay (2006) proposes that the experience of distance and of the exterior localization of percepts requires an active participation of the subject. He has designed a photosensitive device which is attached to one hand and which, upon stimulation with light, gives a vibrating signal at a connected vibrator on the other hand; blindfolded participants are asked to situate light sources in space. With this device, he is able to study “how perceived objects appear in an external space, that is to say, the constitution of a perception space” (Lenay, 2006, p. 29). Lenay (2006, p. 28) comments: “When the device is immobile, (…) the discrimination capacity remains very limited and stimuli are perceived *at the surface of the skin*, but when the device is actively manipulated by the subject, a spectacular capacity of form recognition is observed as well as the exterior locating of the percepts: *the objects observed are perceived in a distal space, over there in front of the subject*.” (Italics added). Strikingly, though the light sources are located at a distance, under specific conditions (namely, immobility of the sensitive cells), they are perceived “at the surface of the skin,” which corresponds literally to what Hervé sometimes describes for targets at a distance. Moreover, his complaint is exactly his inability, at moments, to perceive objects in a distal space “over there in front of” him. In other words, immobilizing the device makes the participant have a perceptual modality which resembles Hervé’s at moments. The coherence between the two situations even increases when Lenay (2006, p. 39) explains: “it is the reversibility, the possibility of returning to the same position which enables the construction of a space of perception.” Hervé “confirms” this need for reversibility not only by his behavior, but also in his stressing of this aspect: “Everything must return to the same.^6^”

#### Indications of reality and efference copies

In the Freudian model of psychosis, processes typical for the unconscious are present in conscious mental life: “As regards the relation of the two psychical systems [the conscious and the unconscious], all observers have been struck by the fact that in schizophrenia a great deal is expressed as being conscious which in the transference neuroses can only be shown to be present in the *Ucs*. [Unconscious] by psycho-analysis.” (Freud, 1915c, p. 197). Lacan (1955–1956, p. 11) also states that “in psychosis the unconscious is at the surface, conscious”. Fenichel (1945, p. 422) agrees: «(…) the impression arises that in schizophrenia “the unconscious has become conscious.”» More specifically, positive psychotic symptoms, such as hallucinations and perceptual distortions, are due to a relative supremacy of so-called “primary processes” versus a downplay of “secondary processes” (Freud, 1900/1958, p. 568; Freud, 1915c, p. 199–204)^7^. Fenichel (1945, p. 422) says literally: « Because the “*primary process*” (…) have come to the fore again, schizophrenics are not estranged by these mechanisms any more.» [Italics added].

Indeed, Freud’s (1950/1966) architecture of his mental apparatus makes use of two kinds of mental processes: primary and secondary processes. Primary process mentation implies a linear reaction upon the characteristics of incoming stimuli aimed at restoring the disturbed equilibrium situation as fast as possible, and therefore characterized by a “flight from the stimulus^8^.” Not all stimuli can be handled in this simple way and especially internal stimuli can not be fled from (e.g., hunger follows you wherever you go). In order to keep alive, an adequate act has to be performed with respect to these stimuli. This adequate act chiefly consists of selecting a specific behavior above other behaviors which therefore must be inhibited; Freud (1900/1958), then, calls this type of mentation the secondary process. For example, a hungry child, having had the experience of being breastfed before, might, upon a new hunger signal, release a sucking behavior (while hallucinating a breast), but if the breast is not in fact present, the hunger will not be relieved and the act will not be adequate (and actually result in a loss of energy). Instead, it might have been more adequate for the child to cry for mother and to wait until a breast is really present before releasing the sucking behavior. In other words, a first challenge in trying to keep alive, is to be able to distinguish a mentally from an effectively present breast and only the secondary process is concerned with this kind of distinctions. Indeed, Freud (1950/1966, p. 325) proposes that for the secondary process to intervene, “it is a question of an indication to distinguish between a perception and a memory (idea).”

Freud (1950/1966, p. 325) then formulates the hypothesis that “it is probably the ω neurons which furnish these indications of reality.” In Freud’s “neuronal” model of the mind, these ω neurons have a particular status. Even though they are “activated along with perception” and “behave like organs of perception” (Freud, 1950/1966, p. 309), they are not, in fact perceptual neurons. Indeed, their discharge direction is *efferent*, i.e., in the direction of motility (Freud, 1950/1966, p. 311). The ω neurons are thus a system of motor neurons which are engaged in the constitution of perception: “it must be assumed that the ω neurons are originally linked anatomically with the paths of conduction from the various sense organs and that they direct their discharges back to the motor apparatuses belonging to those same sense organs.” (Freud, 1950/1966, p. 326). ω neurons, then, could be thought of, for example, oculomotor neurons: they are linked with the sense organ of vision and direct their discharges to the muscles of the eye which enable the precise direction of the gaze. Moreover, Freud (1950/1966, p. 325) indicates: “In the case of every external perception a qualitative excitation occurs in ω […] [this] ω excitation leads to ω discharge, and information of this, as of every discharge, reaches Ψ.” Ψ, then, is a system of cortical neurons with memory capacity responsible for psychical processes in general (Freud, 1950/1966, p. 300). In other words, in the case of external perception, ω will discharge, [eye] movement will be effectively realized by mobilization of the [oculomotor] muscles, *and there will be a central reafferent information of this discharge, i.e., of the efferent command to the eye muscles*. Freud (1950/1966, p. 325) then adds: “***The information of this discharge from ω is thus the indication of quality or of reality for Ψ***.” Since information of the ω discharges is only produced when there is effective, i.e., *active* perception – for example when there is scanning motor activity in the case of vision – this information, the indication of quality or of reality, furnishes a criterion to distinguish external perceptions from internal images, that is, it allows “a discrimination between memory and perception” (Shevrin, 1998, p. 252)^9^. For example, the criterion distinguishes the imagined breast from the perceived breast and only in the latter case, sucking action is released.

**Figure 1:**
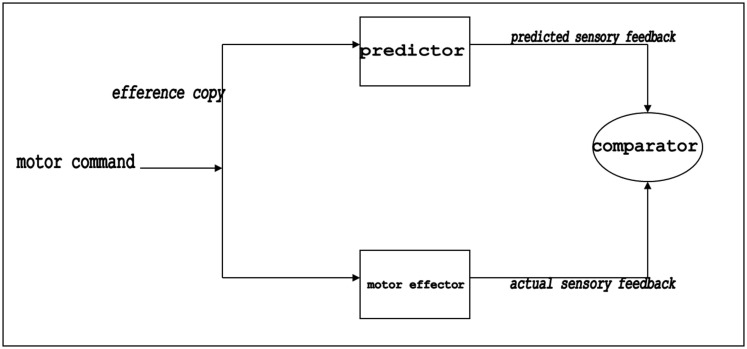
**The efference copy model (Sperry, 1950; von Holst, 1954; Blakemore et al., 1998; Georgieff and Jeannerod, 1998)**. This computational model proposes that upon motor preparation and intention, copies of the efferent motor information are fed back and used centrally in an emulation algorithm, which calculates the anticipated somatosensory changes expected as a consequence of the prescribed motor execution. Upon effective execution, peripheral changes at the level of the muscles, the joints and the skin generate an actual proprioceptive feedback, which will (more or less) balance out the predicted sensory feedback in the (somato-)sensory cortices (at the level of a so-called “comparator”).

Elsewhere (Bazan, 2007a,b; Bazan and Van de Vijver, 2009; Bazan and Snodgrass, 2012) we have defended the idea that these indications of reality are equivalent to the modern notion of “efference copies.” Efference copy models (see Figure 1) are computational models which propose that upon motor preparation and intention, copies of the efferent motor information are fed back and used centrally in an emulation algorithm, which calculates the anticipated somatosensory changes expected as a consequence of the prescribed motor execution (Blakemore et al., 2000). Upon effective execution, the actual proprioceptive feedback of that action will then (more or less) balance out the predicted sensory feedback (at the level of a so-called “comparator”). Thereby, the efference copy is an early-warning signal sent by motor production areas to the corresponding somatosensory areas specialized in the proprioception of this motor execution. Next to the anticipation and the preemptive attenuation, the signal also allows the perceptual structures to distinguish between self-mediated and externally mediated signals.

The modern efference copy models are derived from von Helmholtz (1878/1971, p. 123) original model which first proposed the idea of direct sensation of the motor command: “The impulse to move, which we initiate through the innervation of our motor nerves, is immediately perceptible.” This idea was later integrated in motor physiology as the “corollary discharge” by von Holst (1954) and Sperry (1950), and recently reintegrated in the efference copy model in neurosciences by Blakemore et al. (1998), Wolpert (1997), Jeannerod (1997, 2001), Frith et al. (2000), and others. Freud adhered to the views of the late nineteenth century physiology school – called “physikalischen Physiologie” – of among others, von Helmholtz. The historical, neuro-anatomical, and even semantic closeness^10^ of Freud’s concept of “indications of reality” and the modern sensorimotor concept of “efference copies,” then, is remarkable (see Bazan, 2007a,b for detailed discussion).

#### Hervé as the key between the sensorimotor and the psychodynamic reading

One way to understand Hervé’s problems is to consider that the oculomotor scanning movements are the reversible movements which enable the appearance of percepts in an external space, that is to say, the constitution of a perception space. Indeed, even when fixating, eyes continuously produce movements (Yarbus, 1967; for a recent review Martinez-Conde et al., 2004). Oculomotor fixational mechanisms seem particularly important for moving targets (Skavenski et al., 1979). Eye movements thus support our visual system in the generation of a conscious representation of our external world (Schütz et al., 2011). Hervé does not seem to have a problem with the eye movements *per se*, but, we propose that the changed dynamics are situated at the level of the efference copies of the oculomotor commands.

Three lines of reasoning lead us to this proposition: this would (1) explain Hervé’s different symptoms coherently; (2) fit with a psychodynamic account if we accept an equivalence between efference copies and indications of reality; and (3) fit with a neuroscientific account of other described phenomena in psychosis. In this sense, surprisingly, it is an parsimonious proposition.

First, concerning Hervé’s symptoms. If the efference copies are not functioning “properly,” the pursuit eye movement would still occur, but would not be recognized as being under command of the self. In other words, it would feel as if the eyes were forced by some external agent to make small movements to follow the target, something one could tentatively describe as a “pinching” on the retina, which is what Hervé reports about his experience. Moreover and importantly, if something is disturbed at the level of the efference copies, Hervé is (at moments) without the means to appropriate his own eye movements which gather the retinal information, i.e., he has no means to feel how moving his eyes is efficient in influencing the incoming retinal stimulation. Now, according to the efference copy models^11^, feeling how voluntary (striated) muscle activity is able to influence the incoming stimulation flow is the criterion used by the central nervous system to distinguish external from internal stimulation sources (Gallistel, 1980; Wolpert and Miall, 1996; Poulet and Hedwig, 2006). Hervé probably looses this criterion, or its modalities have changed, and therefore the retinal stimulation is not seen “out there,” but on the surface of his skin, even penetrating his body. In this perspective, it might be interesting to underline that although fixational eye movements have a magnitude that should make them visible to us, we are remarkably unaware of them (Martinez-Conde et al., 2004). Speculatively, though eye movements are voluntary movements, they might be especially well anticipable and therefore their preemptive attenuation through the efference copy system, might be, by default, particularly efficient, rendering us unaware of them, but also contributing to the quality of evidence with which distance or externality of percepts is experienced. It is then, when the efference copy dynamics are possibly disturbed, such as in Hervé’s case, that we come to see that the experience of distance is not an evidence, but the result of an active constitution. Finally, let’s suppose that his eye movements are at particular moments not able to create this experience of distance. As a consequence, we could understand his own back and forth movements, as well as those he asks from others, as a – partially successful – attempt to restore a distance or externality-constituting movement, by replacing a structurally unconscious, low cost mechanism, by another, cognitively decided, and therefore fully conscious and mentally expensive mechanism.

Second, concerning a psychodynamic account of Hervé’s problems. If we would accept the idea of an equivalence between sensorimotor efference copies and Freudian indications of reality, we would be entitled to understand Hervé’s particular perceptual modalities as resulting from a faltering secondary processing of perception, resulting in primary process predominance. (Indeed, as indicated earlier, secondary process mentation requires the use of the indications of reality.) Restated in this approach, Hervé’s perceptual symptoms – though a superficial account of some of them (e.g., the importance of the back and forth movements) might lead to classify them as, e.g., obsessional – become coherent with a diagnosis of psychosis, and thereby with his other psychotic symptoms (e.g., neologisms, delusional constructions, hearing of chatter voices). Moreover, this psychodynamic account helps to make sense of another striking symptom of Hervé, which can not as directly find an explanation in a sensorimotor account, namely his profusion of “undesirable images” upon perceptual intrusion. Indeed, if we consider that it is the primary process, which in Hervé dominates perception, this explains not only his deficit in conferring the incoming visual stimuli the tag of “percept” (the primary process is not concerned with criteria to discriminate the sources of the processed stimuli), but moreover, these incoming stimuli are now preferentially treated in a decontextualized, associative way typical for the primary process. Hence, Hervé produces in an associative way an unrestrained range of memory and fantasy contents^12^ which then have a relatively easy access to consciousness. If the secondary processes were functional, there would be verification that these images do not correspond with reality and their access to consciousness would be restrained; without the selective influence of the secondary process, a direct “window into Hervé’s unconscious” opens up.

Third, concerning a neuroscientific account of other described phenomena in psychosis. The proposition that the efference copy dynamics are altered in Hervé coincides with Frith’s hypothesis (the “defective corollary discharge model for verbal auditory hallucinations”; Frith, 1992; Frith et al., 2000) for the voices heard by psychotic patients. Applied to speech motor dynamics, the efference copy is an early signal sent by the speech production areas to the speech perception areas where it also acts as a criterion to distinguish self-mediated and externally mediated signals. Therefore, a failure at the level of the efference copy can lead to misattribution of inner speech to an external source. In other words, the verbal auditory hallucinations are thought to be due to self-generated subvocal movements^13^ which are not recognized as being self-generated (McGuire et al., 1995). The same hypothesis holds for what Frith (2005) calls the “delusions of control”: due to a difficulty at the level of the efference copy system, psychotic patients are abnormally aware of the sensory consequences of their actions and do not feel in control of their movements. This leads them to believe that their actions are being controlled by an external agent. Another, seemingly anecdotic observation in psychotic patients fits coherently with the model. Blakemore et al. (1998), indeed, have shown that psychotic patients, in contrast with non-psychotic controls, are more sensitive to their own tickling. They ascribe this sensitivity, again, to a possible absence or decrease of the efference copy-mediated attenuation of the proprioceptive consequences of the proper movements in psychosis.

In summary, we have proposed that Hervé’s intrusive experience of the world is to be understood in a psychodynamic framework as due to his psychotic condition and therefore linked to an inability of the secondary process to effectively use the indications of reality as a criterion, and, by consequence, as due to a relative deficit in secondary processing, leading to a dominance of primary process mentation. In Hervé’s case, the primary process dominance is manifest especially in the perceptual modality, leading to a deficit in conferring the incoming visual stimuli the quality of “percept” and treating them preferentially in a decontextualized, associative and fantasmatic way. In parallel, we have proposed that Hervé’s intrusive experience of the world is to be understood in a sensorimotor framework as due to a changed dynamic at the level of the efference copy dynamics of the oculomotor processes when fixating or pursuing moving targets, leading to a deficit in appropriation of these oculomotor movement, which could at the same time explain both Hervé’s pinching feeling on his retina when visual targets move suddenly and his difficulty in experiencing these targets as external or “out there.” Moreover, we have proposed that, in essence, both readings come down to a single common basic mechanism, namely, the ability to distinguish external from internal stimulation, given we use the proposed equivalence between Freudian indications of reality and sensorimotor efference copies as the translation key.

### Denis and the flexible direction of attention

#### Denis

When I meet Denis, he is 45 and he is at the psychiatric center of Beernem^14^. He is the only son of a mother who comes from a big family with origins in France and from a Flemish father. They lived in West-Flanders near the border with France. His mother’s grandmother died under the English bombs in Kortrijk during the last months of the occupation in 1944. Mother is an accountant in her father’s business, specialized in the repair of looms. The company had grown significantly in recent decades. His father, an only son, works in an insurance company. The father’s family are “dead simple” people, some of whom work in the company of his mother’s father. Denis says his troubles start at 15, when he had to learn French. “Je dois” (“I must”) is spelled with a final “s” in French, which is silent: “Why should I write it then? This extra letter is pointless! Maybe once, long ago, it was heard. Language evolves.” When he is 17, his father dies of asphyxiation after a long respiratory illness. He obtains a qualification in electricity. At 20, he stops school and does his military service, after which he is first an apprentice in a mold-making company, and then works for a company of barrels during a year: “That’s when it all started. I was tired, exhausted to death from thinking. I spent all my free time in bed. I fought, fought the obsessive thoughts.” During the next holidays, he feels fit again, parties excessively, and doesn’t sleep. At 24, Denis is admitted to the psychiatric hospital for the first time for a complete lack of sleep and exhaustion. One year later, he attempts suicide with medication: the obsessive thinking drives him desperate. His situation stabilizes again after this, until a close childhood friend, bearing the same first name as his own and who had an identical twin brother, is killed in a car accident in Germany together with his girlfriend. His burial was, according to Denis “very strange.” The childhood friends, regathering after so long, set themselves apart, and begin to share stories from their common past. “I did not feel at ease, I went out with a weird feeling. I didn’t tell anybody. We cried a lot at the funeral. I cried also. I saw a friend crying, crying, crying. Other ones, “hard” ones have cried too. (…) I don’t do graves any more. It doesn’t do me any good, I’m bad for days after that.”

One of Denis’ major difficulties is that often his thoughts impose themselves violently upon him and invade his mental space whether he likes it or not. In session, he reports that when he perceives the world, his mental space is invaded by the details. If he would let himself go, he would be quickly swallowed up by the dots of the wallpaper, the lines in the wood, the irregularities in the carpet, the contour lines of the furniture, etc.: any asymmetry, irregularity, or protrusion would take him completely. These imperfections threaten to invade his whole mental space and lead him from question to question: where does this asymmetry come from, how was it produced, why, what was the intention of the person who produced it^15^?

Denis says that these questions do not really interest him; he is simply taken by them: “You can ask yourself a million of questions if you want and on how many questions will you have answers? The more you know, the more you realize that you do not know. *These obsessive ideas. In fact, they don’t interest me. In fact, they don’t interest me. They enter my mind spontaneously, I can’t help it, and nonetheless they take my attention*.” He then must invest a huge amount of time and energy to *counter-think* a mass of thoughts which would make his life impossible: “The obsessive thoughts take so much energy that you can no longer function, these are thoughts which do not let you go. *Normally, these thoughts are filtered. I see everything. I see a circle on the computer and I wonder: why is that? I can not switch to something else*, it’s not funny. It started when I was 15, all day long at school I was elsewhere with my thoughts. (…) ***I have to force my mind not to be lead by it all the same. I am a fighter, but mentally. This system of counter-computing, I try to apply it to everything now, perhaps with time it will become automatic***, if I’m lucky.» This state of affairs obliges Denis to invest structurally an amount of time in his daily program dedicated to the activity of counter-thinking. He gets so mentally tired of this, that he is no longer able to keep energy for the simple tasks of life or to live an independent life.

#### The flexible direction of attention

There is some resemblance between Denis’ symptoms and the clinical picture of dorsal simultagnosia, a condition caused by a bilateral parietal lesion, where the patient is able to direct his attention to only one single visual element after which his attention remains attached to that. Jeannerod and Jacob (2005, p. 310) consider that the specific difficulty is the disengagement of visual attention: “to engage his visual attention to a novel stimulus, one must first be able to disengage his visual attention of its previous or current allocation. The parietal lobes play a critical role in this attentional mechanism. A bilateral parietal lesion should by consequence lead to attention that “sticks” to the current object without the possibility of turning to another.” Although the clinical pictures are not identical, Denis is also unable to direct attention at will and there is a sticky, or an unbridled, allocation of attention.

The sensorimotor hypothesis for Denis then is that, as in Hervé’s case, his inability to disengage attention is connected to a difficulty involving the efference copy-mechanism. More specifically, the withdrawal of attention would require the attenuating effect brought about by the efference copies. Indeed, say we also consider the allocation of attention as a movement of the subject, then the efference copies not only would yield a way to appropriate the attentional movement (“I choose to pay attention to this”) but also, thanks to their possibility of targeted inhibition^16^, a way to select the contents of attention, i.e., to filter. This hypothesis fits well with the “Corollary Discharge of Attention Movement” (CODAM) model for attention (and consciousness; Taylor, 2003, 2007) which says that the deployment of attention primarily depends on the ability to predict a future state, and that this ability is created by an efference copy of the attention control signal (Taylor and Rogers, 2002). Taylor (2007) proposes that a forward model helps updating the receptive fields of the retina, thereby enabling the switching of attention.

This forward model crucially makes use of efference copies, i.e., copies of the attention movement signal (Taylor, 2007, p. 990) for updating the error in the comparator. Taylor and Fragopanagos (2007, p. 1002) indicate that the efference copy “provides various levels of inhibition of distracters to prevent their access to buffer sites.” The subtraction brought about by the efference copy of the attention movement is probably at the essence of its role in allowing the switching of attention, and thus in preventing distracters to monopolize the mental space. Denis’ description of his experience seems amazingly coherent with this model: “My illness is that there is so much information in my mind that I get stuck in my brain… *I get stuck for one to two seconds and each time I have to re-adjust*… *I have built in an internal system whereby I can think away the obsessive thought in one second*… *when I think of something that is, then I think “stop that!” and so I do not go on*… *in normal people this happens seamlessly*… (…) ***That system of subtraction***, *I try to apply that all over, maybe over the years it will become fluid.”*

Freud (1950/1966, p. 326) also stressed the role of the “information of discharge of the ω-neurons,” this is of the indications of reality, in the allocation mechanism of attention: “the excitation of the ω neurons can also serve to protect the Ψ system (…) by drawing the attention of Ψ to the fact of a perception being present or absent. (…) the information of the latter [ω] discharge (the information of *reflex attention*) will act to Ψ biologically as a signal to send out a quantity of cathexis in the same directions.” In other words, the indices of reality function as a criterion for the flexible investment of mental means. So, again, we find a striking parallel between the Freudian indications of reality and the sensorimotor efference copies. For this reason, the idea is that Denis has an altered dynamic of his indications of reality, and consequently, at the level of the secondary process treatment of attention. The resulting primary process predominance can, moreover, help understand the delusional tendency with which Denis tries to make sense of his experience: « The misery starts with the things made by man, they harass me^17^, as opposed to the things that God made.^18^»

#### Hervé and Denis

For both Hervé and Denis throwing a glance upon the world carries the risk that this world would either invade or overwhelm them. To restore a bearable relation, Hervé has to invest physical means in undoing things and Denis has to invest mental means in counter-thinking things. Denis replaces a structural and unconscious inhibition mechanism for the disengagement of attention by a cognitive inhibition mechanism – the fully conscious decision to stop the allocation of attention and to move on to something else – which requires a continuous mobilization of mental investment. In other words, it seems that according to both models (Freud, sensorimotor) what is at stake in their symptomatology and their suffering, is situated at the level of the *inhibition system*. Both for Hervé and for Denis what is proposed is that their observed relative failure of inhibition would be related with changed dynamics at the level of the indications of reality, and therefore, the secondary process from a psychodynamic point of view and of the efference copies from a sensorimotor perspective. Note that this proposed failing inhibition in psychosis is not in contradiction – and might actually fit quite well – with some of the prevailing neuroscientific theories on schizophrenia which state a diminished inhibitory prefrontal functioning, such as the hypofrontality theory (e.g., Weinberger et al., 1991; Buchsbaum et al., 1992; Schroeder et al., 1994; Volz et al., 1999; Hazlett et al., 2000; Molina et al., 2005; Snitz et al., 2005; Harrison et al., 2006) or the frontotemporal disconnection theory (e.g., Friston and Frith, 1995; Friston, 1998; Fletcher et al., 1999; Lawrie et al., 2002; Crossley et al., 2009).

At this point, let’s dare a further proposition. Indeed, the fundamental metapsychological difference between a neurotic and a psychotic structure is supposed to be the presence, resp. the (occasional) absence of *repression*^19^. The philosopher De Waelhens (1978/2001, p. 149) and the psychoanalysts Laplanche and Pontalis (1973) summarize the evidence in Freud’s texts for this position of the failure of repression in psychosis. Lacan (1955–1956) expresses the psychotic’s situation by saying that the unconscious is exposed for all the world to see (“*a ciel ouvert*”^20^). The proposition therefore is that the failing repression in psychosis then is instantiated as this failing sensorimotor inhibition. In the next case study, we will try to disentangle the same principles applied to language. It is at the level of the (failing) inhibition of language processing that the idea of this inhibition being a form of repression, takes its fullest sense.

### Zac and the repression of language associations

#### Zac

When I meet Zacarie, he is 52 and he is at the psychiatric center of Beernem^21^. He is the third child in a family of six, of whom only he and his elder brother were born in Africa. When he was 3 years old, the family left the country hurriedly to come back to Belgium. He indicates he has no memories of this African childhood, except, as he says, some flashes of “black thoughts” – which he now associates with a preference for black women and for the French language. His father apparently was at the head of a pharmaceutical company in Africa and resumed a veterinary practice once back in Flanders. After a childhood which he calls “fantastic,” a school time that seemed quite average, but without real problems, and a military service time of which no particularities are mentioned, it is then around the age of 20 that he really has difficulties to face life. He doesn’t succeed in managing neither any further education nor training, or in keeping any job or relationship. Instead, he abandons himself in partying, carousal, taking drugs (by injection) and in idleness. Around the age of 26, the first hospitalizations are reported. He accuses his family – especially his mother and sisters – of attempting to poison him. At 33, he is admitted to the psychiatric center. At 40, he is present when his father died in hospital. Poison, the pharmacopeia, intravenous injections, blood transfusion, and euthanasia are the constant elements of his delusional construction^22^.

One of Zac’s major difficulties is that often in an emotionally challenging context, received language fragments “explode” in chains of associative meanings. For example, during one of his visits, the doctor had expressed hope that Zac’s next outing would indeed *take place*. Zac did not understand the wordings of the doctor which also suggested the eventuality of the outing *not* taking place. He suspected malice, and this malice for him was clear and even proved by the doctor’s choice of word: “taking place” – which is “doorgaan” in Dutch. This word “doorgaan” had put Zac in disarray. “Doorgaan,” after all, he tells me, is divided into “door” and “gaan.” “Door” is the inverse of “rood,” meaning “red” and red is the color of blood. “Rood. In vain,” he says, using the English “in vain” and playing on both readings, “in vain” (“hopeless”) but also “in the veins.” “Door hart,” he continues, which means “through the heart” and he clarifies “steek door mijn hart” (“stab through my heart”). The second part of the word “gaan” means “go.” “Gaan” rhymes with “aan.” “Zet maar aan,” he says, which means: “Blow off.” “Aan” is the inverse of “naa” which leads to “naald,” which means “syringe.” In other words, he finally concludes about the doctor’s word, “doorgaan” refers to [twice its reverse] “a red syringe,” and then: “Let me have an injection and that I’d be put in the cell^23^.”

Here is an excerpt of a session on a metonymic rather than an alliterative fashion: “I speak like a boy cow, holy cow, a truth as a cow^24^, the sacred truth. You can know the truth by circling around the pot^25^, but the more you circle around the pot, the more it stinks. If your opponent understands that you’re circling around the pot, then you turn from right to left. The doctor is very good for that. If you speak normally, then you are normal, then you fly to jail^26^. If you act abnormally, then they say “you are abnormal” and you run free. “What’s up?” [they ask]. If it is good, is it reasonable, when things are going less well, it is better. “Has it improved?” [they ask]. Never say “improved,” only “better,” because otherwise… otherwise you’re biting on small fish. Fish such as crab salad and all such things. Little scoundrels^27^. A man in a thousand is a scoundrel, one who fights for his skin. Sometimes fights and battles. There are days when I think of my past, of my father who… I can not let me go, I must continue to fight, fight to achieve something. Left, right, forward, backward, up and down, back and forth. Not gone and not visible. A torture, a martyr. I’ve been through a lot in my life^28^.»

#### The repression of language associations

Psycholinguistic research shows that language is always ambiguous, even for sentences without polysemous words (words with several possible meanings), be it by the simple fact that the pauses in speech taken by the speaker do not match the boundaries of words. The linguists Cutler et al. (2002), for example, indicate that a simple sentence, which is apparently without ambiguities, such as “we stop begging” transiently activates meanings corresponding to intermediate words like “east” (between “we” and “stop”), “top” (in “stop”) and “egg” (in “begging”). Elsewhere we have summarized these results on language ambiguity (Bazan, 2007b, pp. 63–67; Bazan, 2011)^29^. Briefly, for polysemous words, all meanings, even those inappropriate to the context, are activated during a brief time laps of ±100 ms. After this, contextually non-appropriate meanings are actively inhibited and only the selected meaning reaches consciousness. Subliminally presented words also activate both direct semantic associates and the semantics of their *phonological* variants (Klein Villa et al., 2006). These experimental data suggest that incoming language can be considered a stimulus with a high potential to activate interpretations and that without restrictions, the psychic apparatus would be inclined to indulge in an exhaustive spectrum of interpretations of this material including its phonological variants and the semantic associates of these phonological variants. In other words, without inhibition, language is potentially explosive.

This is, of course, what we propose for Zac: when at (emotionally challenging) moments his inhibition system fails or works less well, he has to do with an explosive linguistic stimulus which requires a gigantic interpretation effort from him. Zac’s problems have been classically described for psychotic patients before. Starting from the earliest nosographic descriptions of psychosis, a loosening of language associations has been reported (e.g., Bleuler, 1911). In empirical research, reaction time studies show a more rapid and a wider dispersion of activity in semantic networks of psychotic subjects, which has been described as a disinhibition of the dispersion of automatic activation (Spitzer et al., 1993). Semantic priming tasks, cued word recall and word association tasks all detect increased activation in semantic association networks in subjects with schizophrenia (e.g., Spitzer et al., 1994; Levine et al., 1996; Nestor et al., 1998; Özkarar et al., 2008). Words with remote or indirect connections (such as, e.g., “lemon” and “sweet”) are more easily available to the production and reception language systems in psychosis (Spitzer and Kammer, 1996; Mathalon et al., 2002). Moritz et al. (2001) also observe that the secondary meanings of a word are far more activated in psychotic patients than in controls. They speak of dispersion of activation which is not only faster and wider, but also “more oblique,” and tangentially related with the discourse. The semantic overactivation results have been confirmed with neuro-imaging techniques (e.g., Weisbrod et al., 1998; Hazlett et al., 2000; Higashima et al., 2000; Nohara et al., 2000). For all these reasons, it seems fair to propose that what is problematic in Zac is his ability to repress language associations. Note that this semantic over-associativity has already been interpreted before in a neuropsychoanalytic framework as a failure to repress (see Özkarar et al., 2008).

#### The role of efference copy-induced attenuation in language processing

Language, be it produced, received or thought, always involves an articulatory motor mobilization. Elsewhere, we have summarized the empirical results substantiating this position (Bazan, 2007b, 2011). Briefly, concerning received language, Rizzolatti and Arbib (1998) first described the mirror neuron system in the context of speech perception providing a neurological instantiation for the “motor theory of speech perception” already proposed by the linguists Liberman and colleagues (Liberman et al., 1967; Liberman and Mattingly, 1985). This theory proposes that the linguistic perception of an auditory stimulus necessarily involves the mobilization of the proper linguistic motor circuit^30^. Callan et al. (2003) indicate that reading lips contributes to the understanding of speech through a dynamic of mirror neurons. Moreover, as was demonstrated by brain imaging (Skipper et al., 2007), visual information, transformed into motor information, has a direct impact on the perception of the auditory stimulus. In other words, observing articulatory gestures – e.g., rounding or pressing of the lips – as a visual stimulus in others, and also as a sensorimotor stimulus in oneself (for review see Schwartz et al., 2008), participates directly to the final modulation of the linguistic stimulus heard. In summary, the sensorimotor perception of articulatory movements produces true phonetic information.

Now, there is for the speech movement, as for any other type of movement, an efference copies-mediated attenuation of the somatosensory feedback of the articulatory motor pathways (Heinks-Maldonado et al., 2005; Christoffels et al., 2007) which mutes the hearing of the proper voice. But efference copies also intervene in language perception of others, once the visual or auditory stimulus is transformed into motor information (Skipper et al., 2007). The sensorimotor hypothesis for the case of Zac, then, would be that his inability to repress linguistic associations, would involve an alteration in the efference copy-mediated attenuation of language perception, be this language produced by others or by himself. The idea, then, is that the attenuation of the incoming phonetic signal, at the same time implies the attenuation of its semantic interpretation.

This idea results from the following reasoning. Both our own speech and the speech of others have a considerable level of predictability. However, when something non-expected is said, this is experienced biologically as a failing of our prediction system (i.e., a failing of our movement apparatus) to ward off the incoming signal^31^ and the non-attenuated part of the stimulus will draw attention. The idea with Zac, then, is that, due to changed efference copy dynamics, the on-line attenuation of predictable language fragments fails to some extent, and that, by consequence, the whole incoming language stimulus, to its fullest extent and including its predictable or banal parts, becomes a target of further mental processing. At the same time, with Zac, we also observe that a semantic interpretative counterweight is offered for the whole stimulus. Therefore, we are drawn to infer that the role of the efference copy-induced attenuation of the incoming language signal is also to keep down its all-out interpretation.

In other words, through Zac’s case, we propose that “normally” easily anticipated language is structurally attenuated and that this attenuation stops the reverberation of interpretative activation. For the unanticipated fragments, the non-attenuated, positive somatosensory activation would trigger a mental mobilization, i.e., would lead to the disinhibition of interpretative or identificatory activation^32^. This activation of semantic contents functions as a counterweight to the unexpected part of the language stimulus. Through Zac’s case, we take the measure of the importance to structurally keep the language machine more or less muted: this attenuation prevents the indiscriminate engagement of interpretative activity in favor of those selected fragments on which interpretation is probably the most useful and informative.

#### Primary process language

Zacarie explains how he sees the words line up letter by letter and form geometrical figures. In these trapezoids, he “sees” the letters spin^33^, change order or position, yielding graphemic and phonological variants. Translations of these words in French and English come on top, together with alliteration as well as puns between all these words, their semantics and their common expressions. This makes Zac go literally crazy and, as is the case for Hervé and Denis, he has an astute understanding of his madness: “*Worries in my head, digits and letters in my head, reversed digits and letters, I can’t silence it, everything twists and turns, words, digits, this spinning makes me mad*.” In a psychodynamic framework, this type of language, which is governed by an associative principle of the “anything goes,” is a language on the primary process mode. By contrast, the secondary process allows a symbolic type of language, which is characterized by the fact that the intention of the linguistic act governs its organization (see also Bazan, 2007b). This intention allows an inhibition of associative tendencies in favor of a contextually – or socially – appropriate use of words^34^.

Again, it is striking to notice that this primary process predominance-hypothesis is also what is predicted by the sensorimotor theory, if we accept the premise that copy-efferences and indices of reality are equivalent. Indeed, with altered indices of reality, the secondary process can not function, resulting in primary process dominance. Circumstantial evidence is thus accumulating for this hypothesis of equivalence, which make sense of a large range of different clinical and empirical observations simultaneously with one key supposition.

### Conclusions from psychosis: Repression as the efference copy-mediated attenuation

Taking together our three case studies – having in common an inability to hold something down or out – we propose, as others have done (Frith, 1992, 2005; McGuire et al., 1995; Blakemore et al., 2000; Frith et al., 2000), that a key change for psychosis in its diversity, is at the level of the efference copy system. Moreover we also propose that this neuroscientific reading corresponds with a precise psychoanalytic understanding, implying an equivalence between the sensorimotor efference copies and Freud’s indications of reality. Their faltering role leads, in Freud’s model, to an inability of the secondary process to deploy itself and, thus, to downplay primary processes, resulting in a relative predominance of primary process mentation in psychosis. In line with this, Freud has proposed that repression fails in psychosis and Lacan has taken up this proposition leading to an “unconscious at the surface” (see above). *The proposition therefore is more precisely that the altered efference copy dynamics corresponds with the absence of repression in psychosis*. The strength of this framework is that one key supposition explains a variety of observations in psychosis from both a sensorimotor and a psychodynamic perspective – such as hearing voices, experiencing the world as intrusive, the feeling of being directed by external forces, compulsions of different kinds (to un-think or counter-think, behavioral compulsions) as well as high language associativity.

## The Constitutive Role of Inhibition for Mental Phenomena

The aim of this second part is to check if the application of the hypothesis which we concluded from our psychotic case studies – namely that the attenuation enabled by the efference copy dynamics is, in some instances, the physiological instantiation of the mental mechanism of repression – makes sense in neurosis, where repression is precisely thought to be the main defense mechanism. As the efference copy dynamics refer to motor control regulation, we start this exercise by thinking repression in an action selection framework.

### Inhibition and mental imagery

The moment where something truly mental comes into being can be situated very minutely in Freud’s oeuvre. We propose that it is precisely when the hungry baby, having been (breast-)fed before, is again taken by hunger and cries, but no mother, no breast, or no food comes along and the baby *hallucinates* the coveted object, i.e., has an inner imagery experience independent of the mobilization of his perceptual apparatus.

What actually happens at this point has also been described, independently of Freud, by the neuroscientist Marc Jeannerod. Jeannerod (1994) in the context of motor physiology, indicates that a movement is organized in function of a desired goal, spelled out as a desired final body configuration. For example, a hungry baby might have as a desired goal, a position of the head such that the breast is seen in a certain angle, optimal for the sucking movement to have effect (i.e., for milk coming in). If there is effectively a breast, the sucking movement will be released as soon as the desired and the perceived image coincide, i.e., the baby will turn the head slightly in search for this coincidence. Note that though the desired image is the image of a breast, its physiological instantiation is a motor mobilization for a body configuration optimally able to grasp the breast^35^.

Interestingly, Jeannerod (1994, p. 201) also describes what happens when the movement is *unable* to reach the desired goal, e.g., when there is no breast: « these neurons encode final configurations (of the environment, of the body, of the moving segments, etc.) as they should arise at the end of the action, and (…) they remain active until the requested configuration has been obtained. If the goal [of an action plan] were not reached, the sustained discharge would be interpreted centrally as a pure representational activity and give rise to *mental imagery*.» (Italics added). In other words, to the extent that a movement is not leading to an intended goal, mental imagery arises centrally on the basis of the desired final configuration. For example, this is the principle which explains the phenomenon of phantom limbs. Since no limb is present, there is insisting motor intention while there is a radical absence of motor execution, resulting in a phantom experience. Ramachandran (1994, p. 314) indicates: “the sensations arise from reafference signals derived from the motor commands sent to the phantom,” where “reafference” is to be understood as the efference copies, since they are derived from the motor commands. In the case of E. P., a case described by McGonigle et al. (2002), interestingly, a supernumerary phantom limb is observed, while there was no lost or amputated limb. Indeed, E. P. is a mesofrontal stroke patient, who at moments perceives a supernumerary “ghost” arm. A cluster in the right supplementary motor area (SMA) is mobilized during the presence of the ghost arm. The authors conclude that activation in motor regions of the brain may be sufficient to cause somatic phantom perceptions (McGonigle et al., 2002).

With no breast present, the child turns and reaches out his head unsuccessfully, and this, following Jeannerod, gives rise to mental imagery of the desired final configuration, shaped in function of the breast. In other words, the child will experience a mental image with the meaning of a breast. How can a perceptual experience arise from the sole mobilization of the proper motor circuits? Again, the key model is the efference copy model. Indeed, as described higher (see Figure 1), simulation of action induces an anticipatory attenuation of its expected sensorimotor consequences. In other words, intention, anticipation, preparation, etc. of action yields somatosensory activation, in the absence of any real action execution. When the movement is thereupon perfectly executed, the efference copy-induced preemptive attenuation is exhaustively neutralized by the proprioceptive feedback. At the so-called “comparator” both information cancel each other out and no mental imagery is expected^36^. However, when the anticipated movement is withheld or deviated by either external or internal constraints, to the extent that the realization comes short of exhausting the prediction, there is a non-neutralized attenuation rest, i.e., a rest of negative activation (i.e., inhibition) at the (somato-)sensory cortex. This inhibition rest is the fully sensory result of a movement intention which has not come to (complete) execution. In other words, a true sensory experience has arisen entirely independent of a stimulus input in the perceptual apparatus, on the sole basis of a motor intention. It is striking to notice that Freud (1950/1966, p. 387) had a similar view on the matter when he declares that “motor images are sensory.”

At first, the child has no means to distinguish this mental from a perceived image and the sucking movement will be released. The mental image “feels” as a perceived image, i.e., we have here Freud’s *hallucinatory* wish fulfillment. Later on, maybe due to the maturing efference copy-pathways of the oculomotor movements of vision, the child has the means to distinguish perceived from internally generated images. Indeed, the movements of the eyes will have different – namely, more dramatic – effects for a perceived than for an internally generated image. At this stage, if no breast is present, the baby will still generate an internal image of the breast, but this image will be recognized as a mental image, and is thus no longer a hallucination. At the same time, as the internal origin of the mental image is recognized, the sucking movement is withheld. The Swiss psychiatrists Saraga and Gasser (2005, p. 111) indicate that Freud underscored the importance of this inhibition as being the essence of the secondary process, which enables the development of thought itself, the “substitute of the hallucinatory wish fulfillment.” We might say that in the case of an imagined breast, the sucking action “is not hypercathected, remains thereafter in the Ucs.” (Freud, 1915c, p. 202), that is, we have here a very basic form of repression (namely of the motor act of sucking). Indeed, the French psychoanalyst Le Guen (2001, p. 46) underscores that “what has to be inhibited in fact not the object, but truly the motor act, as a function.”

In summary, modern neuroscience proposes that mental imagery is in its nature close to action, and emerges when action is withheld, interrupted or frustrated. This is coherent with Freud’s view on the matter of 1895.

### Inhibition and mental reality

Probes from the outer (and inner) world continuously activate (historically) associated action plans. We are not conscious of these activations, but they can be demonstrated experimentally (see subliminal motor priming research: e.g., Schlaghecken and Eimer, 2002; Jaskowski, 2007, 2008; Boy et al., 2008; Boy and Sumner, 2010). For directed action, there needs to be a continuous selection of one alternative with inhibition of the others (e.g., Nachev et al., 2007). The word “inhibition” then refers to the withholding of an action avenue to proceed to execution. If the action is not executed, the preemptive attenuation will not be compensated, and this will result in a negative activation, i.e., an inhibition at the level of the sensory cortex. “Inhibition” in this second instance then means a lowering of the function (cf. Freud, 1926, p. 87). So the prevention of an action to be executed, leads to the *status quo* of the sensory inhibition and this (somato-)sensory inhibition would constitute the source of the mental imagery^37^.

In other words, the first moment of action priming leads to exhaustive activation, i.e., to the priming of (potentially) all action alternatives associated to the stimulus and available to the particular subject. In a second time, one action choice is selected by inhibition of the other alternatives. Consciousness of the stimulus, and of the reaction upon it, would only arise in this second time, and even – as proposed by Haggard and Eimer (1999) – *as a result* of this selective inhibition. By contrast, the inhibited action plans normally do not become conscious.

To understand how this selection happens, we again suggest a Freudian and a sensorimotor approach. Freud (1950/1966) explains that in order to obtain an “identity of thought” between a wishful image and a perceptual image, facilitated associations have to be interrupted by what he calls “side-cathexis”. The facilitated associations, indeed, are the stimulus-driven associations, and they are not directed toward a goal. Freud (1950/1966, pp. 376–378) explains how it is possible to think from a perceptual image toward a wished-for goal: “Here [in the case of practical thinking] a wishful cathexis is firmly retained, while alongside of it a second, perceptual, cathexis which emerges is followed with attention. (…). The trend toward going in the direction of the best facilitation will, however, be interrupted by the presence of *side*-*cathexis*. Supposing that three pathways lead from *a*, to *b*, *c*, and *d* (their [amount of] facilitation being in that order), and that *d* lies in the neighborhood of the wishful cathexis +*V*, then the consequence may be that the *Q*Φ [quantity from the system Φ, the perception system], in spite of the facilitations, will flow not to *c* and *b* but to *d*, and from there to +*V*; and thus the pathway *W–a–d–*+*V* will be revealed as the one that is being sought. Here there is in operation the principle which we have long recognized that cathexis can divert facilitation and can thus operate against it, and that accordingly a side-cathexis modifies the passage of *Q*η. (…) The aim of practical thought is *identity* (…) the need for thought thereupon ceases and that, instead, a full innervation is permitted of the *motor images* touched upon on the path, which represent what is in the circumstances a justified accessory part of the *specific action*^38^.” We suggest that pathways *b*, *c*, and *d* are action alternatives elicited by the perceptual stimulus and/or by activation *a*. It will be in function of the wishful result, and by means of its cathexis, that *b* and *c* will be “inhibited” (i.e., its facilitation diverted) in favor of the selected pathway *d*. Contrary to Maze’s (1983) criticism that the inhibition by side-cathexes is a teleological mechanism, we have here a framework which *mechanically* explains targeted action selection, by using the wishful image as a reference for practical thought. Moreover, if the side-cathexis is a way to inhibit the activation of certain pathways, we have here the description of what is known in sensorimotor terms as “reverse engineering” or “inverse models” (e.g., Pouget and Snyder, 2000; Tin and Poon, 2005): with the wished-for final configuration or goal kept in mind, it is computed “backward” which motor steps would be required to get there (for an overview, Flanders, 2011; Gordon et al., 2011). In other words, an “inverse model” starts from a desired goal and generates a motor command which attempts to achieve that goal. This motor command is thereupon put through a forward model, which calculates the expected outcomes on the basis of the efference copies. Selection happens through mutual inhibitory connections between response alternatives (see Sumner et al., 2007). Praamstra and Seiss (2005) propose that alternating cycles of activation and inhibition are inherent in the competitive interactions between response alternatives, perhaps due to a mechanism that detects and opposes large activation differences. Moreover, Sumner et al. (2007) demonstrate that certain motor areas (the supplementary eye fields and the SMA) are critically involved in this unconscious suppression of unwanted responses elicited by the surrounding context.

Thus, inhibition is the condition for targeted action. But, even if there is exhaustive activation in a subject, the pattern of activations is still determined by the particular way that subject’s memory is organized. By consequence, targeting an action toward a precise goal, induces inhibition of a specific pattern of action alternatives, and therefore – since action inhibition induces mental imagery – it also induces a range of mental images, specific to each subject. By consequence, the way in which a subject comes to execute a particular action might differ only from the way in which another subject comes to execute that exact same action by the action alternatives he had to inhibit in order to make this choice. What we propose, then, is that the (continuously) induced unconscious mental images by the mere fact of acting in the world, constitute a mental life with a concrete instantiation, the physiology of which has been spelled out by e.g., Jeannerod (1994). These unconscious mental images then constitute in concreto what Freud (1900/1958, p. 613) calls the *psychic reality*: “The unconscious is the psychic itself and its essential reality.” In other words, the difference between two subjects, performing the same behavior, might be limited to the difference in unconscious mental reality which performing this behavior has induced.

Directed action in the world, and the structural inhibition this requires, would thus continuously induce an (unconscious) mental reality, specific to each subject. What evidence is there for this supposed structural inhibition? The principal indications are indirect, and primarily the fact that inhibition betrays itself by its spill-over effects, i.e., by being too performing and inhibiting elements which are thereupon needed. For example, in the case of action selection, Sumner et al. (2007, p. 699) indicate: “On occasions, such automatic [inhibition] mechanisms might appear maladaptive, suppressing actions that are subsequently required.” As debated higher, the linguistic act is also a motor act. Gernsbacher and Robertson (1995) indicate how this spill-over is also observable in language dynamics^39^. For examples, participants who had to understand the following sentence ending upon a polysemous word “he lit the *match*” and were immediately thereupon presented a sentence where an alternative meaning of the same word is needed: “he won the *match*,” have a measurable delay in disambiguating the second sentence. This is explained by the supposition that upon the first hearing of the word “match,” all the meanings of the word are first transiently activated, whereupon the meaning of “lighter/fire” is contextually selected and all the non-contextual alternative meanings (e.g., game, peer or equal etc.) are inhibited. If one of these inhibited alternatives by coincidence is thereupon required, activating this meaning costs more since there is a recent inhibition that still has to be overcome. Note that there is some similarity between this dynamic and what Freud (1901/1978, pp. 2–7) has described for the forgetting of the name “Signorelli.” Indeed, Freud explains his (temporary) inability to find the name of the Italian painter “Signorelli” by the fact that shortly before this moment, upon debating the manners of the Turcs with his recently met travel companion, a sentence had come to his mind “*Herr*, was ist da zu sagen” (“Sir, what can I say?”) which he had withheld himself from sharing with his companion since it referred to inappropriate sexual content. He did so well in withholding this, that he also inhibited the associated semantic and phonological variants, including the Italian translation of “Herr,” *Signor* and the phonologically associated *Signorelli*. Also note that this dynamic, in Freud’s thinking, is an instance of repression.

In summary, voluntary behavior implies a potential for coactivation of action plans. The actual action pathway is computed through an inverse model going backward from a desired goal and alternative action plans are inhibited. This inhibition sometimes betrays itself through its spill-over effects, rendering previously inhibited action avenues momentarily unavailable while they are needed. This was already Freud’s view on the matter in 1895 and 1901. We propose to add to this that the inhibited action alternatives supposedly induce mental images, which constitute an unconscious mental reality, different for each subject.

### Inhibition and repression

Now, not all action or meaning alternatives upon a probe are equivalent in their probability or in their strength of being associatively activated, in within the same subject. Action or speech fragments with a higher emotional valence pop up more easily, while at the same time, often, their execution is not contextually appropriate. In some cases, their execution might even lead to a threateningly dangerous level of emotional mobilization of the inner body. But, as Freud (1915c, p. 178) states, “to suppress the development of affect is the true aim of repression and […] its work is incomplete if this aim is not achieved.” As their motor plans are highly invested with intentionality and at the same time their execution has to be very much prevented^40^, these highly emotional fragments require a more structural level of inhibition, as compared to the on-line routine inhibition for action or language alternatives which are not specifically emotionally charged. Freud speaks in this context of “anti-cathexes” or “counter-cathexes (Gegenbesetzungen): “[Since] the repressed exercises a continuous pressure in the direction of the conscious… this pressure must be balanced by an unceasing counter-pressure” (Freud, 1915b, p. 151; cf. Freud, 1926, p. 157). Shevrin (1990, p. 105) agrees that if we are to explain repression then this “more or less permanent counterforce must be conjectured to exist.” Repression, then, is considered a special instance of inhibition for emotionally threatening stimuli.

At the sensorimotor level, the enduring absence of execution implies an important generation of efference copy-induced somatosensory attenuation, with a systematic absence of compensation of this anticipatory inhibition. As a consequence, these action or language fragments are particularly prone to induce mental imagery, without the subject being necessarily conscious of this. This relationship between repression and motor representation has also been underscored by the metapsychological reading of Le Guen (2001, pp. 45, 46, 61): “that a motor action (or, at least, a *representation* *of this motor action*) would be inherent to repression is a long established fact”; “to prevent efficiently the expression of motor expression, the part that represses has to ‘*represent*’ this motor discharge” and “repression is a necessary condition for the establishment and the function of *motor representations*” [Italics added].

So, at the one hand, inhibition mechanisms are structural for any targeted action or for normal language understanding and they are thought to induce representational activity or mental imagery through an efference copy-mechanism. At the other hand, due to this need for inhibitory selection, some emotional alternatives have to be submitted to a stronger form of inhibition. ***The degree of inhibition might determine the type of motor imagery induced***. At the one hand, we propose that *representations* arise as the result of motor intention *not exhaustively met* by motor execution, i.e., in the gap between intended and executed movement. Therefore, they arise as a consequence of “normal” on-line action selection and inhibition in the course of targeting behavior. At the other hand, we propose that *phantoms* arise as the result of sustained motor activation which is systematically not met at all by any actual execution. Therefore, *the species of mental imagery induced by this more radical inhibition, characteristic for repression, is thought to be of the “phantom”-typ*e.

Speculatively, representations, due to the partial success of their cancelation – which indicates that the organism has at least partially understood how to fight them off, i.e., partially successfully identified the stimulus – are associated with semantic activations, while phantoms, due to the total absence of any cancelation, are pure motor forms. Elsewhere (Bazan, 2007a, 2011) we have defended the idea of “phantoms,” especially “phonemic phantoms.” Briefly, phonemic phantoms are word or language fragments which are especially mentally invested, due to their existential importance to the subject, but which have to be kept under repression, to avoid the excessive development of affect. This results in motor phantoms with the phonemic form of the repressed fragment, occupying the mental space with mental preoccupation. As the conscious thoughts concerning the fragments are most probably unbearable, and by means of the polysemy of language, preoccupation (as well as symptoms) will arise concerning homophonic equivalents of the fragments, see e.g., Freud (1900/1958, p. 596): “The ideas which transfer their intensities to each other stand in the loosest mutual relations. They are linked by associations of a kind that is scorned by our normal thinking and relegated to the use of jokes. In particular, we find associations based on homonyms and verbal similarities treated as equal in value to the rest.” This is what Lacan (1957/1966) has called the signifier. Indeed, the phonemic phantom has only a motor articulatory form; it does not in the first place specify semantics. Therefore, motor expression of the mental investment can be realized through speaking and/or acting upon the homophonic equivalents. It is through these signifier-effects (both mental preoccupation and symptoms) that a hint can be read concerning what is truly at stake mentally. But as these substitutes have no real impact on the origin of the unrest or discontent, no real relief can be expected from these signifier symptoms.

The structural inhibition on emotional fragments thus induces (1) mental imagery with formal characteristics of the repressed fragment, i.e., phantoms, which result at the level of the psyche in mental preoccupation and symptoms (especially, symptoms with a signifier-marked form) and (2) spill-over effects which result in the return-of-the-repressed (such as e.g., forgetting of names and words, parapraxes, behavior tendencies etc.). Therefore the proposed model for the physiological instantiation of repression, with as a key supposition that the efference copy-induced attenuation of motor intentions is totally left unanswered, in order to radically prevent execution of an action which would lead to development of excess affect, yields the mental phenomena characteristic for neurosis (mental preoccupation, symptoms, and return-of-the-repressed). In addition, we have shown that not only in its results, but also in its dynamics, the sensorimotor model corresponds well with what Freud had already proposed starting from his *Project* in 1895. Note that this hypothesis also fits well with one of the main neuropsychoanalytic understandings of repression reviewed by Boag (2007, 2012), namely an inhibitory account with respect to competing responses and response selection.

## Conclusion

In a first stage, going minutely through three clinical case studies of psychotic patients cross-facing the psychodynamic and the sensorimotor data, has resulted in the proposition of an equivalence between the Freudian indices of reality and the sensorimotor efference copies. Though at the level of different psychological functions, our three cases of psychosis share as a common mechanism a failure of inhibition (at some level and at some moments). We propose to understand this failure of inhibition as equivalent to the psychodynamic understanding of the failure of repression in psychosis. Therefore, we end up with the idea that the attenuation enabled by the efference copy dynamics is, in some instances, the physiological instantiation of the mental mechanism of repression.

In a second stage, we have applied this conclusion to the way mental processes are thought to arise, both according to sensorimotor and to Freudian views. The efference copy model is very much at the heart of this proposition; it is the one key mechanism that connects action with a sensory experience without perceptual input and which probably explains the closeness between action and representation. Jeannerod’s idea that sustained activation of an action goal, without reaching it, would give rise to mental imagery, is a second key in the proposed model. Indeed, it links inhibition of motor intentions to mental imagery. As a consequence, as inhibition mechanisms are structural for any targeted action or for normal language understanding, targeted action or disambiguated language processing are thought to structurally induce mental imagery, constituting a subjective unconscious mental reality.

Repression, then, is considered a special instance of inhibition for emotionally threatening stimuli. Due to the characteristics of this inhibition, which leaves the motor intentions totally unanswered, it is proposed to yield a specific type of motor imagery, namely “phantoms,” which are in the first place characterized by their form. These phantoms are thought to induce mental preoccupation, as well as symptoms (of everyday life) which especially through their form are thought to refer to the repressed motor fragment. This, then, is especially true for the articulatory form of language, yielding symptoms characterized by the signifier in their form.

Going back to psychosis at this point, we propose that there is probably routine sensorimotor inhibition in psychosis, such as there is in neurosis, but that the process is structurally less stable in psychosis. Clinics show us that it is less stable in some modalities in particular (perception, language, attention etc.) depending on the subject, but next to this modality-specific instability, it might also be less stable in general, since we frequently find (isolated) language, attention, motor, perception etc. disinhibition symptoms in psychotic patients who do no necessarily display dominant symptoms in these respective modalities. In other words, even if some modality may be touched more specifically, the process in itself is less stable. Since repression is defined as a special instance of inhibition for emotionally threatening stimuli, i.e., structurally requiring even stronger than normal inhibition, it makes sense that this process then is structurally failing in psychosis.

## Conflict of Interest Statement

The author declares that the research was conducted in the absence of any commercial or financial relationships that could be construed as a potential conflict of interest.

## Footnotes

^1^Through Schreber’s words, I want to thank these middle-aged men, whose daily world is mostly limited to the large corridors of the psychiatric centre, Hervé, Denis, Zacarie, *and others*, who have given me a privileged access to their enlightenment. Notes from observations from 26.09.2005 till 21.08.2007.

^2^See also Van de Vijver et al. (2006). Hervé is referred to in this text as R.V.

^3^For more details on this case study, see “Excerpts from the Case Study of Hervé” in Appendix.

^4^And which he has graciously lend me to take a copy. Hervé knows that I am writing papers mentioning his case (he has asked me directly). Notwithstanding I left the psychiatric centre in 2007, I keep calling him on the phone once every 2 weeks until this day to hear how he is doing. Now and then he inquires about my work and if he appears in it.

^5^Original version see “Excerpts from the Case Study of Hervé” in Appendix.

^6^Moreover, his delusional constructions try to make sense of this need for reversibility, see “Hervé” in Appendix.

^7^Using a simple pen-and-paper probe for primary and secondary process mentation designed by Brakel et al. (2000) at the University of Michigan (the “GeoCat”), we have found empirical evidence strengthening this proposition (Bazan et al., 2012).

^8^In its simplest form, imagine the amoeba which, upon receiving light stimuli on its membrane surface, will react linearly by opposing to this – threatening – increase in potential energy the movement of its flagella, as a result of which it flees away from the light source and diminishes its energy level or its tension. This tension relaxation making the potential energy level returning to baseline, then, is the “pleasure principle” in its simplest form.

^9^Shevrin (1998, p. 137) even proposes more generally that “The function of consciousness as subjective awareness in any state is to distinguish the actual source of a mental content and to reveal the source as a quality or category of experience,” these categories referring to mental vehicles such as perception, memory, thought, desire, and so on. He says the indications of reality thus give “tags” to the mental contents.

^10^Between “Abfuhrnachricht” (literally: efference message) and “efference copy.”

^11^As it is to Freud (1915a, pp. 119, 134): « On the one hand, it [the organism] will be aware of stimuli which can be avoided by muscular action (flight); these it ascribes to an external world. On the other hand, it will also be aware of stimuli against which such action is of no avail and whose character of constant pressure persists in spite of it; these stimuli are signs of an internal world, the evidence of instinctual [drive] needs. The perceptual substance of the living organism will thus have found in the efficacy of its muscular activity a basis for distinguishing between ‘outside’ and ‘inside’.” and “The antithesis ego-non-ego (external), i.e. subject-object, is […] thrust upon the individual organism at an early stage, by the experience that it can silence *external* stimuli by means of muscular action but it is defenceless against *instinctual* [drive] stimuli” (see also Van de Vijver, 2000).

^12^There is some analogy between the way Hervé produces these mental images as counterweight for the perceptual intrusion and the way the amoeba produces flagella movements as counterweight for the light stimulation – in this sense both are primary process “flights for the stimulus.”

^13^Indeed, Séglas (1892) had already noticed that the hallucinatory experience of voices in psychotic subjects is accompanied by a proper subvocally articulation. In 1981, Green and Preston (1981) record the verbal hallucinations of a patient with two microphones placed on either side of his larynx. The electromyographic recording of the speech muscles shows increased activity during hallucinations. Brain imaging studies show that auditory hallucination in psychosis correlates with subvocal speech (e.g., Bick and Kinsbourne, 1987; Liddle et al., 1992).

^14^See also Van de Vijver et al. (2006). Denis is referred to in this text as C. V.

^15^Though there is a potential attentional threat coming from all things, his main fixations seem to be in the visual field, see Section “Excerpts from the Case Study of Denis” in Appendix.

^16^Since this is the first occurrence of the word “inhibition,” I want to stress that, unless otherwise indicated, the term “inhibition” is used in its physiological or sensorimotor definition, namely, a process through which action is cancelled or suppressed, and not in its Freudian sense (though, as discussed further in Inhibition and Mental Reality. the two concepts are also logically related).

^17^“Die doen me de duivel aan,” litterally “they do me the devil.”

^18^Indeed, in the “harassment”-complaint a paranoid allusion may be heard and in the word-play devil/God an allusion to a delusional construct. That this was only the discrete tip of the iceberg, would become clear later on, when Denis opened up and, generously, agreed to share some of his elaborate delusional constructions.

^19^In a Lacanian perspective (Lacan, 1955–1956), a psychotic structure is characterized by another defence mechanism, called *rejection*. The metapsychological difference between repression and rejection is that in repression there has been a time 1 where the representation was mentally accepted (integrated) before it was isolated from the associative chain, i.e., repressed. In rejection there has never been a time where the representation was integrated in associative chains: it remained “at the periphery” of the mental apparatus, and it is there where it returns (e.g. hallucinations at the perceptual periphery, motor behaviours “directed from outside” at the motor periphery).

^20^The failure of the repression in the Lacanian approach is the failure of the paternal metaphor : “It is in an accident in this register [signifying chain i.e. language] and in what takes place in it namely, the foreclosure of the name of the father in the place of the Other, and in the failure of the paternal metaphor, that I designate the defect that gives psychosis its essential condition, and the structure that separates it from neurosis.” (Lacan, 1977, p. 238).

^21^See also Van de Vijver et al. (2006). Zacarie is referred to in this text as L. D. Z.

^22^He blames his intimates of attempting to poison to him; he has already resigned to this “fact” and therefore demands euthanasia, preferably by blood transfusion with animal blood. My work with him mainly consists of listening, during which I try to cling to the twists of his associations. I sometimes stop him and ask him for clarification over certain connections. During our 2 years of work together Zacaria evolved from a rather depressed and apathetic mood to a dynamic and active mode of life. He first started to collect music, and then engaged in a musical band at the psychiatric center in which he sings. He then had quite regular performances with this band in Flanders and beyond. However, the subjective experience he reports all through during sessions, invariably was on the complaint mode, an accusation that he has been, he is and he will be wronged. At the moment of the writing of this paper, he has even succeeded in leaving the psychiatric hospital and lives on his own in his apartment.

^23^In other words, and with some free interpretation from my side: “What the doctor actually meant is that he wanted to get rid of me by stabbing my heart with a syringe of blood [or something of the kind]. If that’s the case, let me have euthanasia by injection right away – and since people here don’t believe my delusions and don’t tolerate my accusing behaviour, that I’d be put in cell right away.” For another excerpt, see “Excerpts from the Case Study of Zac” in Appendix.

^24^A “a truth as a cow,” Dutch expression for “an evident truth.”

^25^“Circle around the pot,” Dutch expression meaning “beat around the bush.”

^26^The Dutch word for jail is “nor” which is phonologically close to “normal.”

^27^The Dutch word for “scoundrel” is “crapuul,” which is phonologically close to “crab” in its first syllable.

^28^For the original Flemish version, see “Excerpts from the Case Study of Zac” in Appendix.

^29^Here are some of the important references: Swinney (1979), Onifer and Swinney (1981), Seidenberg et al. (1982), Simpson and Burgess (1985), Gernsbacher and Faust (1991), Paul et al. (1992), Simpson and Kang (1994), Gernsbacher and Robertson (1995), Faust and Gernsbacher (1996), Gorfein et al. (2000).

^30^Note that this was already Freud’s proposition in comparable wordings in his study on aphasia (Freud, 1891/1953, pp. 91–92): “Understanding of spoken words is probably not to be regarded as simple transmission from the acoustic elements to the object association; it rather seems that in listening to speech for understanding, the function of verbal association is stimulated from the acoustic elements at the same time, so that we more or less repeat ourselves the words heard, thus supporting our understanding with the help of kinaesthetic impressions. A higher measure of attention in listening will entail a higher degree of transmission of speech heard on to the tract serving the motor execution of language.”

^31^i.e. “the flight for the stimulus” has failed.

^32^The mechanism has similar dynamical principles as for the hungry child: the information from the motor system had indicated that the image of the breast is a perception, i.e., there is really a breast, and the sucking motor activity is released, disinhibited.

^33^Notice that language perception might require, at a first (normally unconscious) level, a reversible back-and-forth (articulation) movement, in the same way Lenay (2006) has suggested that the constitution of a visual perception requires a reversible back-and-forth (oculomotor) movement.

^34^While the intention is given directly in the wordings of the neurotic subject (with full employment of the grammatical form “I [verb]”), the intention is frequently given in an allusive way in the speech of a psychotic subject.

^35^This is not that different from how e.g. the neuroscientists Grabowski et al. (1998) and Grafton et al. (1997) conceive of the way objects are coded in the brain: indeed, they indicate that object and action observation, most prominently tool (use) observation – and to a lesser extent voiced object and action naming – all seem to activate premotor circuits that would be involved in the actual use of the object. Grafton et al. (1997, p. 235) comment: “To categorize an object, it is not enough to have a description of its visual characteristics; it is necessary also to understand its use. The premotor activations may subserve the motoric aspects of object semantics.” In other words, objects are encoded as the motor program which we have to mobilise to use these objects – i.e., as the body configuration optimally able to grasp or take hold of these objects. In the present case, the object “breast” is (also) encoded as a motor program which has to be mobilised to be able to grasp the breast.

^36^cf. Freud (1915b, p. 153): « The general vicissitude which overtakes the idea that represents the instinct can hardly be anything else than that it should *vanish* from the conscious if it was previously conscious, or that it should be held back from consciousness if it was about to become conscious. » [Italics added].

^37^In other words, inhibition of an action choice, in the sense of preventing it to go to execution – this prevention not coming from within the movement, but (as explained below) working inversely from a movement goal backwards – leads to a status quo of the somatosensory inhibition. In other words still, and confusingly, inhibition leads to (maintaining) inhibition.

^38^The specific action or adequate act is, in Freud’s model, the act that will be able to relieve the tension, i.e. which will be able to satisfy the need, which is the origin of the drive leading to the act.

^39^The same spill-over principle is described in memory research: Anderson and Green (2001) and Conway (2001) have shown that the choice for a precise form is made at the expense of the inhibition of other competing form candidates.

^40^Indeed, Freud (1915b, p. 157), Freud (1915c, p. 179), Freud (1926, p. 95) says: “[repression] entails abstention from action, a motor fettering of the impulse”; “repression results not only in withholding things from consciousness, but also in preventing the development of affect and the setting-off of muscular activity” and again “repression displays its power (…) The substitutive process is prevented, if possible, from finding discharge through motility (…). It must not be transformed into action.” Le Guen even refers in this context to what Freud (1926, pp. 119–120) has called the defensive technique of “undoing”: “the neurotic person will try to make the past itself non-existent. He will try to repress it by motor means. (…) We thus unexpectedly discover *a new, motor technique of defence*, or (as we may say in this case with less inaccuracy) *of repression*.” [Italics added].

## Appendix

### Excerpts from the case study of Hervé

Hervé: “Since my sister was also handicapped, she was the first “kouwekouwe.” She was mentally handicapped. She also took pills. She looked into the air and she trembled. Her finger was stiff, her leg moved constantly. (…) She pulled out her pants, she sat with her bare buttocks, she squatted. (…) The neighbors came to tell us that she was exposing her buttocks again at the street side and mother had to go get her. (…) It was the sexual drive, she wanted to get laid.” (28.11.2005).

Hervé would give names of his own to certain things, persons, or phenomena (neologisms). *Kouwekouwes* in his vocabulary refer to people who look straight ahead, are aggressive and regularly smash all things to pieces; they are in contrast with *Oetkers* who are stupid people who do things awrily wrong, therefore are unpredictable, and therefore they make him angry. The word “kouwekouwes” stems from when Hervé was 10, from music or a song on the Flemish radio and also: “My sister sat in that car, my father’s car. It was a bin on a harness that squeaked: *squeak squeak* when he turned, because the suspension was blown, therefore we made a kouwekouwe car of it.” (24.11.05). The word “Oetkers” comes from a firm of proteins, chocolate, spaghetti, foods: “Mother had brought a pot of chocolate spread when I was 13, chocolate to prepare my sandwiches. There was a teddy bear on the pot that winks an eye to the cat besides him, that it is good, that the food is tasty and they have no more chocolate spread to eat. Even an elephant, a hare. I have made “Oetker” of that by looking at it and ponder on it that I’m not an Oetker, that I don’t want to be pushed by someone else.” (19.09.05).

Original version in Flemish of Hervé’s note on pages 2–3 (all names were replaced by their initials): “11-09-1995. In Dymfna. Die vuile vetvest, kelderlapjes. Een naald in mijn oog. Ontbinding van mijn fotoapparaat als G. C. aan de kast passeerde in de eerste living. Mijn oog uitzuigen. 28-11-‘95 Een penis door mijn mes. 28-11-1995. Mijn borstspier gescheurd als P. L. in de gang passeerde. Rond die tijd overgeplaatst in naar St. Cornelius. Mijn ingewanden uitgehaald door C. C. aan de vroegere wasserij op 21-12-’97. 21-11-98 Als de ergotherapeute zich naar de boekbinderij begaf, Lieve C. i.p.v. Koen C. Als J. D. van de plaats waar de opener gang naar zijn stoel ging. Mijn kloten afgeschoeperd of afgesmolten. Als de brandweercommandant aan de boerderij ging. Mijn dikke darm wat opengescheurd. Als M. D. van de W. C. naar buiten ging. Mijn kloten uitgehaald.”

Hervé: “To undo everything up to my 12 years – or else, to undo everything completely, to undo my whole life, back to zero. What if the universe would return to zero? I would have to start again, in the way it would please God, forward and backward. Is that God’s will? That God be the master of the universe, he would resurrect men, that is my idea. [We would] come back with a blank sheet, start fornicating again, start working again – for example firefighter, plumber. I have to pay for the sins of another, of reincarnation, how a man is reincarnated, for the sins of my grandfather, or for other sins which I am not.” (29.11.2005). Or another time: “to go back again, to go back again, to undo things. All that has happened in the world, to return with a clean slate. [to where?] I don’t know, I don’t know to what point I would want to go back. Actually I would like the very universe to have to go back. When they go to the toilet, the shit back in the intestines, the sandwiches back on the plate, the farmer the spuds back into the ground. The whole creation undone, I ponder that it should be so. [Why?] Because I had too many bad thoughts in the past.” (03.11.2005).

### Excerpt from the case study of denis

Denis: « In secondary school, we were taught optics in the physics classes. Concave lenses, convex lenses, concave-convex lenses, convex-convex lenses, … inverted image, virtual image, … Why do these spots shine and why is there no shine elsewhere? (…) I look around and I see for example that computer. Then I think: what was the man who made the first computer thinking? How did he come to these thoughts? What is the source of everything? Why? What moved that man? What were the thoughts which lead him to make it as it is? Why are our fingers of different lengths? Why aren’t our toes all of the same length?» (19.10.05).

### Excerpts from the case study of Zac

Here is another excerpt of Zac’s word associations, and its approximate translation. A team member had asked Zac if he would be missing her (during her anticipated prolonged absence). [Again, one might say, there was “malice” in this question of the team member, since how was Zac supposed to answer, given he might have felt sympathy toward her, but he was also resentful against the limitations imposed on him by the team.] He commented upon this question in session with me, giving the reply he would state to her:

« Missen is wissen is wis is wijs is wegwijs
To miss is to erase is wis is wise is oriented
[The word “missen” refers to “wissen” because the letter “w” is a letter “m” upside-down; “wissen,” “wis” and “wijs” refer to each other because of their phonological closeness and “wijs” metonymically refers to “wegwijs”.]
weg (is gew) is gewapend
gone (is “gew”) is armed
[The word “weg” refers to the word “gewapend” by way of the (unsaid) “gew” which
is the phonological inverse of “weg” and the first syllable of “gewapend”]
gewapend (is weg) (is vaarwel) is vaarweg
armed (is gone) (is farewell) is fairway
[The word “gewapend” refers to the word “vaarweg” by way of the (unsaid) intermediaries “weg”, the inverse of the first syllable “gew”, which leads to its compound word “vaarweg”. “Vaarweg” does not exist but is close, both in phonology and in meaning, to the existing word “vaarwel,” meaning “farewell.”]
is weg is weg in ‘t hoofd
is gone is gone in the head
hij is weg in ’t hoofd»
*he’s gone in the head/he has lost his mind*.

Here is the original Flemish version of the excerpt on pages 13–14: « Ik spreek als een boy cow, de heilige koe, waarheid als een koe, de heilige waarheid. Ge kunt de waarheid weten door rond te pot te draaien, maar hoe meer ge rond de pot draait, hoe meer dat het stinkt. Als uw tegenstander door heeft dat ge rond de pot draait, dan draait ge van rechts naar links. De dokter is vree slim daarin. Als ge normaal spreekt, dan zijt ge normaal, dan vliegt ge in de nor. Als ge abnormaal doet, dan zeggen ze “ge zijt abnormaal” en loopt ge los. “Hoe is ‘t?” [vragen ze]. Als ‘t goed is, is ‘t redelijk, als ‘t minder goed is, is ‘t beter.”Is ‘t verbeterd?” [vragen ze]. Nooit “verbeterd” zeggen, alleen “beter,” want anders …dan zijt ge visjes aan ‘t bijten. Vis zoals krabsalade en al zo’n dingen. Crapuultjes. Een man uit de duizend is een crapuul, één die vecht voor zijn vel. Soms die vecht en strijdt. Sommige dagen moet ik denken aan mijn verleden, aan mij vader die… Ik mag me niet laten gaan, ik moet blijven strijden, strijden om iets te bereiken. Links, rechts, voorwaarts, achterwaarts, op en neer, weg en weer. Niet weg en niet te zien. Een foltering, een marteling die ik veel in mijn leven heb meegemaakt.».

## References

[B1] Anderson M. C. Green C. (2001). Suppressing unwanted memories by executive control. Nature 410, 366–369 10.1038/35066572 11268212

[B2] Bazan A. (2007a). “An attempt towards an integrative comparison of psychoanalytical and sensorimotor control theories of action,” in Attention and Performance XXII, eds Haggard P. Rossetti Y. Kawato M. (New York: Oxford University Press), 319–338

[B3] Bazan A. (2007b). Des fantômes dans la voix. Une hypothèse neuropsychanalytique sur la structure de l’inconscient. Collection Voix Psychanalytiques. Montréal: Editions Liber

[B4] Bazan A. (2011). Phantoms in the voice. A neuropsychoanalytic hypothesis on the structure of the unconscious. Neuropsychoanalysis 13, 161–176

[B5] Bazan A. Snodgrass M. (2012). “On unconscious inhibition: instanciating repression in the brain,” in Trends in Neuropsychoanalysis: Psychology, Psychoanalysis and Cognitive Neuroscience in Dialogue, eds Fotopoulou A. Pfaff D. W. Conway E. M. (Oxford: Oxford University Press), 307–337

[B6] Bazan A. Van de Vijver G. (2009). “L’Objet d’une science neuro-psychanalytique. Questions épistémologiques et mise à l’épreuve,” in Vers Une Neuropsychanalyse? eds Ouss L. Golse B. Georgieff N. Widlöcher D. (Paris: Odile Jacob), 33–54

[B7] Bazan A. Van Draege K. De Kock L. Brakel L. A. W. Geerardyn F. Shevrin H. (2012). Empirical evidence for freud’s theory of primary process mentation in acute psychosis. Psychoanal. Psychol. 10.1037/a002713922844181

[B8] Bick P. A. Kinsbourne M. (1987). Auditory hallucinations and subvocal speech in schizophrenic patient. Am. J. Psychiatry 144, 222–225 3812794 10.1176/ajp.144.2.222

[B9] Blakemore S.-J. Wolpert D. M. Frith C. D. (1998). Central cancellation of self-produced tickle sensations. Nat. Neurosci. 1, 635–640 10.1038/2870 10196573

[B10] Blakemore S.-J. Wolpert D. M. Frith C. D. (2000). Why can’t you tickle yourself? Neuroreport 11, 11–15 10.1097/00001756-200008030-00001 10943682

[B11] Bleuler E. (1911). Dementia praecox oder die Gruppe der Schizophrenien. Leipzig: Deuticke 10.1192/bjp.149.5.661 3545358

[B12] Boag S. (2007). ‘Real processes’ and the explanatory status of repression and inhibition. Philos. Psychol. 20, 375–392 10.1080/09515080701361173

[B13] Boag S. (2012). Freudian Repression, the Unconscious, and the Dynamics of Inhibition. London: Karnac, 167–184

[B14] Boy F. Clarke K. Sumner P. (2008). Mask stimulus triggers inhibition in subliminal visuomotor priming. Exp. Brain Res. 190, 111–116 10.1007/s00221-008-1515-5 18682922

[B15] Boy F. Sumner P. (2010). Tight coupling between positive and reversed priming in the masked prime paradigm. J. Exp. Psychol. Hum. Percept. Perform. 36, 892–905 10.1037/a0017173 20695707 PMC3124756

[B16] Brakel L. Kleinsorge M. Snodgrass M. Shevrin H. (2000). The primary process and the unconscious: experimental evidence supporting two psychoanalytic presuppositions. Int. J. Psychoanal. 81, 553–569 10.1516/0020757001599951 10967775

[B17] Buchsbaum M. S. Haier R. J. Potkin S. G. Nuechterlein K. Bracha S. Katz M. (1992). Frontostriatal disorder of cerebralmetabolism in never-medicated schizophrenics. Arch. Gen. Psychiatry 49, 935–942 10.1001/archpsyc.1992.01820120023005 1360198

[B18] Callan D. E. Jones J. A. Munhall K. Callan A. M. Kroos C. Vatikiotis-Bateson E. (2003). Neural processes underlying perceptual enhancement by visual speech gestures. Neuroreport 14, 2213–2218 10.1097/00001756-200312020-00016 14625450

[B19] Christoffels I. K. Formisano E. Schiller N. O. (2007). The neural correlates of verbal feedback processing: an fMRI study employing overt speech. Hum. Brain Mapp. 28, 868–879 10.1002/hbm.20315 17266104 PMC6871445

[B20] Conway M. A. (2001). Repression revisited. Nature 410, 319–320 10.1038/35066672 11268191

[B21] Crossley N. A. Mechelli A. Fusar-Poli P. Broome M. R. Matthiasson P. Johns L. C. (2009). Superior temporal lobe dysfunction and frontotemporal dysconnectivity in subjects at risk of psychosis and in first-episode psychosis. Hum. Brain Mapp. 30, 4129–4137 10.1002/hbm.20834 19530219 PMC6870945

[B22] Cutler A. Demuth K. McQueen J. M. (2002). Universality versus language-specificity in listening to running speech. Psychol. Sci. 13, 258–262 10.1111/1467-9280.00447 12009047

[B23] De Waelhens A. (1978/2001). “The accession to primal repression and language. Their failure in schizophrenia,” in Phenomenology and Lacan on Schizophrenia: After the Decade of the Brain, eds De Waelhens A. Ver Eecke W. (Leuven: University of Louvain Press), 143–174

[B24] Faust M. E. Gernsbacher M. A. (1996). Cerebral mechanisms for suppression of inappropriate information during sentence comprehension. Brain Lang. 53, 234–259 10.1006/brln.1996.0046 8726535 PMC4426501

[B25] Fenichel O. (1945). The Psychoanalytic Theory of Neurosis. New York: Norton

[B26] Flanders M. (2011). What is the biological basis of sensorimotor integration? Biol. Cybern. 104, 1–8 21287354 10.1007/s00422-011-0419-9 PMC3154729

[B27] Fletcher P. McKennab P. J. Friston K. J. Frith C. D. Dolan R. J. (1999). Abnormal cingulate modulation of fronto-temporal connectivity in schizophrenia. Neuroimage 9, 337–342 10.1006/nimg.1998.0411 10075903

[B28] Freud S. (1915a). “Instincts and their vicissitudes,” in The Standard Edition of the Complete Psychological Works of Sigmund Freud, Vol. XIV, ed. Strachey J. (London: Hogarth Press), 117–140

[B29] Freud S. (1915b). “Repression,” in The Standard Edition of the Complete Psychological Works of Sigmund Freud, Vol. XIV, ed. Strachey J. (London: Hogarth Press), 141–158

[B30] Freud S. (1915c). “The unconscious,” in The Standard Edition of the Complete Psychological Works of Sigmund Freud, Vol. XIV, ed. Strachey J. (London: Hogarth Press), 159–215

[B31] Freud S. (1926). “Inhibitions, symptoms and anxiety,” in The Standard Edition of the Complete Psychological Works of Sigmund Freud, Vol. XX, ed. Strachey J. (London: Hogarth Press), 77–175

[B32] Freud S. (1891/1953). On Aphasia, trans. Stengel E. New York: International Universities Press

[B33] Freud S. (1900/1958). “The interpretation of dreams,” in The Standard Edition of the Complete Psychological Works of Sigmund Freud, Vol. IV–V, ed. Strachey J. (London: Hogarth Press), 339–627

[B34] Freud S. (1901/1978). “The psychopathology of everyday life,” in The Standard Edition of the Complete Psychological Works of Sigmund Freud, Vol. VI, ed. Strachey J. (London: Hogarth Press), 1–279

[B35] Freud S. (1950/1966). “Project for a scientific psychology,” in The Standard Edition of the Complete Psychological Works of Sigmund Freud, Vol. I, ed. Strachey J. (London: Hogarth Press), 281–392

[B36] Friston K. J. (1998). The disconnection hypothesis. Schizophr. Res. 30, 115–125 10.1016/S0920-9964(97)00140-0 9549774

[B37] Friston K. J. Frith C. D. (1995). Schizophrenia: a disconnection syndrome? Clin. Neurosci. 3, 89–97 7583624

[B38] Frith C. (2005). The self in action: lessons from delusions of control. Conscious. Cogn. 14, 752–770 10.1016/j.concog.2005.04.006 16098765

[B39] Frith C. Blakemore S. J. Wolpert D. M. (2000). Explaining the symptoms of schizophrenia: abnormalities in the awareness of action. Brain Res. Rev. 31, 357–363 10.1016/S0165-0173(99)00052-1 10719163

[B40] Frith C. D. (1992). The Neuropsychology of Schizophrenia. Hove: Erlbaum

[B41] Gallistel C. R. (1980). The Organization of Action: A New Synthesis. Hillsdale: Lawrence Erlbaum Associates, 166–209

[B42] Georgieff N. Jeannerod M. (1998). Beyond consciousness of external reality: a “who” system for consciousness of action and self-consciousness. Conscious. Cogn. 7, 465–477 10.1006/ccog.1998.0367 9787056

[B43] Gernsbacher M. A. Faust M. E. (1991). The mechanism of suppression: a component of general comprehension skill. J. Exp. Psychol. Learn. Mem. Cogn. 17, 245–262 10.1037/0278-7393.17.2.245 1827830 PMC4311900

[B44] Gernsbacher M. A. Robertson R. R. W. (1995). Reading skill and suppression revisited. Psychol. Sci. 6, 165–169 10.1111/j.1467-9280.1995.tb00326.x PMC4191834 25309047

[B45] Gordon G. Kaplan D. M. Lankow B. Little D. Y. Sherwin J. Suter B. A. (2011). Towards an integrated approach to perception and action: conference report and future directions. Front. Syst. Neurosci. 5:20 10.3389/fnsys.2011.00020 21541257 PMC3083716

[B46] Gorfein D. S. Berger S. Bubka A. (2000). The selection of homograph meaning: word association when context changes. Mem. Cognit. 28, 766–773 10.3758/BF03198411 10983450

[B47] Grabowski T. J. Damasio H. Damasio A. R. (1998). Premotor and prefrontal correlates of category-related lexical retrieval. Neuroimage 7, 232–243 10.1006/nimg.1998.0324 9597664

[B48] Grafton S. T. Fadiga L. Arbib M. A. Rizzolatti G. (1997). Premotor cortex activation during observation and naming of familiar tools. Neuroimage 6, 231–236 10.1006/nimg.1997.0293 9417966

[B49] Green P. Preston M. (1981). Reinforcement of vocal correlates of auditory feedback: a case study. Br. J. Psychiatry 139, 204–208 10.1192/bjp.139.3.204 7317701

[B50] Haggard P. Eimer M. (1999). On the relation between brain potentials and the awareness of voluntary movements. Exp. Brain Res. 126, 128–133 10.1007/s002210050722 10333013

[B51] Harrison B. J. Yücel M. Shaw M. Brewer W. J. Nathan P. J. Strother S. C. (2006). Dysfunction of dorsolateral prefrontal cortex in antipsychotic-naïve schizophreniform psychosis. Psychiatry Res 148, 23–31 10.1016/j.pscychresns.2006.02.006 17052898

[B52] Hazlett E. A. Buchsbaum M. S. Jeu L. A. Nenadic I. Fleischman M. B. Shihabuddin L. (2000). Hypofrontality in unmedicated schizophrenia patients studied with PET during performance of a serial verbal learning task. Schizophr. Res. 43, 33–46 10.1016/S0920-9964(99)00178-4 10828413

[B53] Heinks-Maldonado T. H. Mathalon D. H. Gray M. Ford M. J. (2005). Fine-tuning of auditory cortex during speech production. Psychophysiology 42, 180–190 10.1111/j.1469-8986.2005.00272.x 15787855

[B54] Higashima M. Kawasaki Y. Urata K. Sakai N. Nagasawa T. Koshino Y. (2000). Regional CBF in male schizophrenic patients performing an auditory discrimination task. Schizophr. Res. 42, 29–39 10.1016/S0920-9964(99)00094-8 10706983

[B55] Jaskowski P. (2007). The effect of nonmasking distractors on the priming of motor responses. J. Exp. Psychol. Hum. Percept. Perform. 33, 456–468 10.1037/0096-1523.33.2.456 17469979

[B56] Jaskowski P. (2008). The negative compatibility effect with non-masking flankers. A case for a mask-triggered inhibition hypothesis. Conscious. Cogn. 17, 765–777 10.1016/j.concog.2007.12.002 18226925

[B57] Jeannerod M. (1994). The representing brain: neural correlates of motor intention and imagery. Behav. Brain Sci. 17, 187–245 10.1017/S0140525X00034026

[B58] Jeannerod M. (1997). The Cognitive Neuroscience of Action. Oxford: Black-well

[B59] Jeannerod M. (2001). Neural simulation of action: a unifying mechanism for motor cognition. Neuroimage 14, 103–109 10.1006/nimg.2001.0832 11373140

[B60] Jeannerod M. Jacob P. (2005). Visual cognition: a new look at the two-visual systems model. Neuropsychologia 43, 301–312 10.1016/j.neuropsychologia.2004.11.016 15707914

[B61] Klein Villa K. Shevrin H. Snodgrass M. Bazan A. Brakel L. A. W. (2006). Testing Freud’s hypothesis that word forms and word meanings are functionally distinct in the unconscious: subliminal primary process cognition and its links to personality. Neuro-Psychoanalysis 8, 117–137

[B62] Lacan J. (1977). “On a question preliminary to any possible treatment of psychosis,” in Ecrits: A Selection, trans. Sheridan A. (London: Tavistock), 238

[B63] Lacan J. (1955–1956). “The seminar of Jacques Lacan, book III,” in The Psychoses, ed. Miller J.-A., trans. Grigg R. [1993] (New York: Norton).

[B64] Lacan J. (1957/1966). “The agency of the letter in the unconscious or reason since Freud,” in Ecrits: A Selection, ed. Norton W. W. trans. Sheridan A. [1977] (New York: W. W. Norton), 146–178

[B65] Laplanche J. Pontalis J. B. (1973). “The language of psycho-analysis,” in The International Psycho-Analytical Library, Vol. 94, trans. Nicholson-Smith D. [1994] (London: The Hogarth Press and the Institute of Psycho-Analysis), 1–497

[B66] Lawrie S. M. Buechel C. Whalley H. C. Frith C. D. Friston K. J. Johnstone E. C. (2002). Reduced frontotemporal functional connectivity in schizophrenia associated with auditory hallucinations. Biol. Psychiatry 51, 1008–1011 10.1016/S0006-3223(02)01316-1 12062886

[B67] Le Guen C. (2001). Quelque chose manque.… De la répression aux représentations motrices. Revue Française de Psychanalyse 65, 37–70 10.3917/rfp.651.0037

[B68] Lenay C. (2006). Enaction, externalisme et suppléance perceptive. Intellectica 43, 27–52

[B69] Levine J. Schild K. Kimhi R. Schreiber G. (1996). Word associative production in affective versus schizophrenic psychoses. Psychopathology 29, 7–13 10.1159/000284966 8711078

[B70] Liberman A. M. Cooper F. S. Shankweiler D. P. Studdert-Kennedy M. (1967). Perception of the speech code. Psychol. Rev. 74, 431–461 10.1037/h0020279 4170865

[B71] Liberman A. M. Mattingly I. G. (1985). The motor theory of speech perception revised. Cognition 21, 1–36 10.1016/0010-0277(85)90021-6 4075760

[B72] Liddle P. F. Friston K. J. Frith C. D. Jones T. Hirsch S. R. Frackowiak R. S. J. (1992). Patterns of regional cerebral blood flow in schizophrenia. Br. J. Psychiatry 160, 179–186 10.1192/bjp.160.2.179 1540757

[B73] Martinez-Conde S. Macknik S. L. Hubel D. H. (2004). The role of fixational eye movements in visual perception. Nat. Rev. Neurosci. 5, 229–240 10.1038/nrg1297 14976522

[B74] Mathalon D. H. Faustman W. O. Ford J. M. (2002). N400 and automatic semantic processing abnormalities in patients with schizophrenia. Arch. Gen. Psychiatry 59, 641–648 10.1001/archpsyc.59.7.641 12090817

[B75] Maze J. R. (1983). The Meaning of Behaviour. London: Allen & Unwin

[B76] McGonigle D. J. Hänninen R. Salenius S. Hari R. Frackowiak R. S. J. Frith C. D. (2002). Whose arm is it anyway? An FMRI case study of supernumerary phantom limb. Brain 125, 1265–1274 10.1093/brain/awf139 12023315

[B77] McGuire P. K. Silbersweig D. A. Wright I. Murray R. M. David A. S. Frackowiak R. S. (1995). Abnormal monitoring of inner speech: a physiological basis for auditory hallucinations. Lancet 346, 596–600 10.1016/S0140-6736(95)91435-8 7651003

[B78] Molina V. Sanz J. Reig S. Martínez R. Sarramea F. Luque R. (2005). Hypofrontality in men with first-episode psychosis. Br. J. Psychiatry 186, 203–208 10.1192/bjp.186.3.203 15738500

[B79] Moritz S. Mersmann K. Kloss M. Jacobsen D. Andresen B. Krausz M. (2001). Enhanced semantic priming in thought-disordered schizophrenic patients using a word pronunciation task. Schizophr. Res. 48, 301–305 10.1016/S0920-9964(00)00057-8 11295382

[B80] Nachev P. Wydell H. O’Neill K. Husain M. Kennard C. (2007). The role of the pre-supplementary motor area in the control of action. Neuroimage 36, 155–163 10.1016/j.neuroimage.2007.03.034 PMC2648723 17499162

[B81] Nestor P. G. Akdag S. J. O’Donnell B. F. Ninikiewicz M. Law S. Shenton M. E. (1998). Word recall in schizophrenia: a connectionist model. Am. J. Psychiatry 155, 1685–1690 9842776 10.1176/ajp.155.12.1685

[B82] Nohara S. Suzuki M. Kurachi M. Yamashita I. Matsui M. Seto H. (2000). Neural correlates of memory organization deficit in schizophrenia. Schizophr. Res. 42, 209–222 10.1016/S0920-9964(99)00131-0 10785579

[B83] Onifer W. Swinney D. A. (1981). Accessing lexical ambiguities during sentence comprehension: effects of frequency of meaning and contextual bias. Mem. Cognit. 9, 225–236 10.3758/BF03196957

[B84] O’Regan J. K. Noe A. (2001). A sensorimotor account of vision and visual consciousness. Behav. Brain Sci. 24, Cambridge University Press Available at: http://www.bbsonline.org/Preprints/ORegan/10.1017/s0140525x01000115 12239892

[B85] Özkarar F. G. Göktepe E. Canbeyli R. (2008). Ego fails to repress: the role of left frontal lobe hypoactivation in associative memory impairment in schizophrenia. Neuro-Psychoanalysis 10, 189–199

[B86] Paul S. T. Kellas G. Martin M. Clark M. B. (1992). Influence of contextual features on the activation of ambiguous word meanings. J. Exp. Psychol. Learn. Mem. Cogn. 18, 703–717 10.1037/0278-7393.18.4.703 1385611

[B87] Pouget A. Snyder L. H. (2000). Computational approaches to sensorimotor transformations. Nat. Neurosci. 3, 1192–1198 10.1038/81469 11127837

[B88] Poulet J. F. A. Hedwig B. (2006). New insights into corollary discharges mediated by identified neural pathways. Trends Neurosci. 30, 14–21 10.1016/j.tins.2006.11.005 17137642

[B89] Praamstra P. Seiss E. (2005). The neurophysiology of response competition: motor cortex activation and inhibition following subliminal response priming. J. Cogn. Neurosci. 17, 483–484 10.1162/0898929053279513 15814007

[B90] Ramachandran V. S. (1994). Phantom limbs, neglect syndromes, repressed memories, and freudian psychology. Int. Rev. Neurobiol. 37, 291–333 10.1016/S0074-7742(08)60254-8 7883483

[B91] Rizzolatti G. Arbib M. A. (1998). Language within our grasp. Trends Neurosci. 21, 188–194 10.1016/S0166-2236(98)01260-0 9610880

[B92] Saraga M. Gasser J. (2005). Epreuve de réalité et psychose chez Freud. La fin de la psychose à l’heure d’un dernier « retour»? Psychothérapies 2, 109–115

[B93] Schlaghecken F. Eimer M. (2002). Motor activation with and without inhibition: evidence for a threshold mechanism in motor control. Percept. Psychophys. 64, 148–162 10.3758/BF03194564 11916298

[B94] Schreber D. P. (1903). Memoirs of My Nervous Illness, eds and trans. MacAlpine I. Hunter R. [1988] (Cambridge: Harvard University Press).

[B95] Schroeder J. Buchsbaum M. S. Siegel B. V. Geider F. J. Haier R. J. Lohr J. (1994). Patterns of cortical activity in schizophrenia. Psychol. Med. 24, 947–955 10.1017/S0033291700029032 7892362

[B96] Schütz A. C. Braun D. I. Gegenfurtner K. R. (2011). Eye movements and perception: a selective review. J. Vis. 11, 1–30 10.1167/11.3.1 21917784

[B97] Schwartz J.-L. Sato M. Fadiga L. (2008). The common language of speech perception and action: a neurocognitive perspective. Revue Française de Linguistique Appliquée XIII, 9–22

[B98] Séglas J. (1892). Les troubles du langage chez les aliénés. Paris: Rueff

[B99] Seidenberg M. S. Tanenhaus M. K. Leiman J. M. Bienkowski M. (1982). Automatic access of the meanings of ambiguous words in context: some limitations of knowledge-based processing. Cogn. Psychol. 14, 489–537 10.1016/0010-0285(82)90017-2

[B100] Shevrin H. (1990). “Subliminal perception and repression,” in Repression & Dissociation: Implications for Personality Theory, Psychopathology, & Health, ed. Singer J. L. (Chicago: University of Chicago Press), 103–119

[B101] Shevrin H. (1998). “Why do we need to be conscious? A psychoanalytic answer,” in Advanced Personality, Chap. 10, eds Barone D. F. Hersen M. VanHasselt V. B. (New York: Plenum Press), 239–260

[B102] Simpson G. B. Burgess C. (1985). Activation and selection processes in the recognition of ambiguous words. J. Exp. Psychol. Hum. Percept. Perform. 11, 28–39 10.1037/0096-1523.11.1.28

[B103] Simpson G. B. Kang H. (1994). “Inhibitory processes in the recognition of homograph meaning,” in Inhibitory Processes in Attention, Memory, and Language, eds Dagenbach D. Carr T. H. (San Diego: Academic Press), 359–381

[B104] Skavenski A. A. Hansen R. M. Steinman R. M. Winterson B. J. (1979). Quality of retinal image stabilization during small natural and artificial body rotations in man. Vision Res. 19, 675–683 10.1016/0042-6989(79)90243-8 547477

[B105] Skipper J. I. Van Wassenhove V. Nusbaum H. C. Small S. L. (2007). Hearing lips and seeing voices: how cortical areas supporting speech production mediate audiovisual speech perception. Cereb. Cortex 17, 2387–2399 10.1093/cercor/bhl147 17218482 PMC2896890

[B106] Snitz B. E. MacDonald A. III Cohen J. D. Cho R. Y. Becker T. Carter C. S. (2005). Lateral and medial hypofrontality in first-episode schizophrenia: functional activity in a medication-naive state and effects of short-term atypical antipsychotic treatment. Am. J. Psychiatry 162, 2322–2329 10.1176/appi.ajp.162.12.2322 16330597

[B107] Sperry R. W. (1950). Neural basis of the spontaneous optokinetic response produced by visual inversion. J. Comp. Physiol. Psychol. 43, 482–489 10.1037/h0055479 14794830

[B108] Spitzer M. Hermle L. Maier S. (1993). Associative semantic network dysfunction in thought-disordered schizophrenic patients: direct evidence from indirect semantic priming. Biol. Psychiatry 34, 864–877 10.1016/0006-3223(93)90054-H 8110913

[B109] Spitzer M. Kammer T. (1996). Combining neuroscience research methods in psychopathology. Curr. Opin. Psychiatr. 9, 352–363 10.1097/00001504-199609000-00012

[B110] Spitzer M. Weisker I. Winter M. Maier S. Hermle L. Maher B. A. (1994). Semantic and phonological priming in schizophrenia. J. Abnorm. Psychol. 103, 485–494 10.1037/0021-843X.103.3.485 7930048

[B111] Sumner P. Nachev P. Morris P. Peters A. M. Jackson S. R. Kennard C. (2007). Human medial frontal cortex mediates unconscious inhibition of voluntary action. Neuron 54, 697–711 10.1016/j.neuron.2007.05.016 17553420 PMC1890004

[B112] Swinney D. A. (1979). Lexical access during sentence comprehension: (re)consideration of context effects. J. Verbal Learn. Verbal Behav. 18, 645–659 10.1016/S0022-5371(79)90284-6

[B113] Taylor J. G. (2003). Paying attention to consciousness. Prog. Neurobiol. 71, 305–335 10.1016/j.pneurobio.2003.10.002 14698766

[B114] Taylor J. G. (2007). CODAM: a neural network model of consciousness. Neural Netw. 20, 983–999 10.1016/j.neunet.2007.09.003 17935944

[B115] Taylor J. G. Fragopanagos N. (2007). Resolving some confusions over attention and consciousness. Neural Netw. 20, 993–1003 10.1016/j.neunet.2007.09.003 17935946

[B116] Taylor J. G. Rogers M. (2002). A control model of the movemement of attention. Neural Netw. 15, 309–326 10.1016/S0893-6080(02)00024-2 12125887

[B117] Tin C. Poon C. S. (2005). Internal models in sensorimotor integration: perspectives from adaptive control theory. J. Neural Eng. 2, 147–163 10.1088/1741-2560/2/3/S01 PMC2263077 16135881

[B118] Van de Vijver G. (2000). Identification and psychic closure: a dynamic structuralist approach of the psyche. Ann. N. Y. Acad. Sci. 901, 1–12 10.1111/j.1749-6632.2000.tb06260.x 10818552

[B119] Van de Vijver G. Bazan A. Rottiers F. Gilbert J. (2006). Enactivisme et internalisme: de l’ontologie à la clinique. Intellectica 43, 93–103

[B120] von Helmholtz H. (1878/1971). “The facts of perception,” in Selected Writings of Hermann von Helmholtz, ed. Kahl R. (Middletown, CT: Wesleyan University Press), 366–408

[B121] von Holst E. (1954). Relations between the central nervous system and the peripheral organs. Br. J. Anim. Behav. 2, 89–94 10.1016/S0950-5601(54)80044-X

[B122] Volz H. Gaser C. Häger F. Rzanny R. Poenisch J. Mentzel H. J. (1999). Decreased frontal activation in schizophrenics during stimulation with the continuous performance test – a functional magnetic resonance imaging study. Eur. Psychiatry 14, 17–24 10.1016/S0924-9338(99)80711-1 10572321

[B123] Weinberger D. R. Berman K. F. Daniel D. G. (1991). “Prefrontal cortex dysfunction in schizophrenia,” in Frontal Lobe Function and Dysfunction, ed. Levin H. S. Eisenberg H. M. Benton A. L. (New York: Oxford University Press), 275–287

[B124] Weisbrod M. Maier S. Harig S. Himmelsbach U. Spitzer M. (1998). Lateralised semantic and indirect semantic priming effects in people with schizophrenia. Br. J. Psychiatry 172, 142–146 10.1192/bjp.172.2.142 9519066

[B125] Wolpert D. M. (1997). Computational approaches to motor control. Trends Cogn. Sci. (Regul. Ed.) 1, 209–216 10.1016/S1364-6613(97)01070-X 21223909

[B126] Wolpert D. M. Miall R. C. (1996). Forward models for physiological motor control. Neural Netw. 9, 1265–1279 10.1016/S0893-6080(96)00035-4 12662535

[B127] Yarbus A. L. (1967). Eye Movements and Vision. New York: Plenum

